# Exergy-guided multiphysics analysis of transport irreversibilities in PEMFC flow-field architectures

**DOI:** 10.3389/fchem.2026.1884122

**Published:** 2026-08-06

**Authors:** Saad Alrwashdeh

**Affiliations:** Marine Engineering Technology, Sharjah Maritime Academy, Sharjah, United Arab Emirates

**Keywords:** exergy analysis, flow field design, multiphysics modelling, PEMFC, transport irreversibilities

## Abstract

Transport-induced thermodynamic irreversibilities remain one of the primary factors limiting the efficiency and high-load performance of proton exchange membrane fuel cells (PEMFCs). However, the complex interactions among flow-field architecture, coupled transport phenomena, electrochemical reactions, and exergy destruction are not yet fully understood. This study presents an exergy-guided three-dimensional multiphysics framework that integrates electrochemical kinetics, multicomponent species transport, heat transfer, two-phase flow, membrane hydration, and entropy-generation analysis to investigate transport irreversibilities in hierarchical PEMFC flow-field architectures. The developed framework establishes direct quantitative relationships between flow-field geometry, local transport behavior, and exergy destruction mechanisms under realistic operating conditions. The results show that total exergy destruction increases from 0.14 to 1.10 W cm^-2^ as current density increases, while exergy efficiency decreases by approximately 40%–50% because of intensified mass-transport limitations. The optimized multiscale flow-field architecture improves net power density by up to 19% and exergy efficiency by 24%, while simultaneously reducing pressure losses by 20%–25% and increasing oxygen concentration by 6%–8% compared with the conventional design. Furthermore, transport-related irreversibilities become dominant above approximately 1.5 A cm^-2^, where mass-transfer resistance and viscous dissipation together account for more than 30%–35% of the total exergy destruction. These findings demonstrate that the proposed exergy-guided multiphysics framework provides a physically consistent strategy for identifying dominant transport-loss mechanisms and optimizing PEMFC flow-field architectures for enhanced electrochemical and thermodynamic performance.

## 1 Introduction

The world energy environment is experiencing a paradigm shift with the necessity to cut down greenhouse gases and enhance sustainability over the long term. The traditional fossil-based systems though still prevalent are becoming increasingly restricted by environmental laws and the scarcity of resources. This in turn has seen renewable energy technologies including solar, wind and hydrogen-based systems receiving much attention as a viable alternative. The intermittency of primary renewable sources, however, creates problems of reliability and energy storage, which requires effective energy conversion devices capable of functioning in changing conditions. Hydrogen in this context has been found to be a versatile energy carrier that can be used to bridge between generation and demand, especially when combined with electrochemical conversion technologies (e.g., Hydrogen economy). The strategic role of hydrogen to facilitate low-carbon energy infrastructures has been highlighted by recent research (Bakır and Ağbulut, 2026; Biancolli et al., 2020; Hasan et al., 2026; Wang and Chen, 2026).

Fuel cells are some of the technologies available to convert chemical energy to electricity without combustion, thus providing a direct and efficient route to converting chemical energy into electricity (Gasteiger et al., 2004; Gostick et al., 2009; Hasan et al., 2026; Hu et al., 2026). Their efficiency, low emissions, and scalability have brought about a wide interest within the transportation, stationary power, and marine sectors. Fuel cell systems are being investigated to be incorporated in electric vehicles, distributed generation, and supplementary systems. A study by Tien, et al. has pointed out the practical benefits of fuel cells (in respect of modularity and operational flexibility). More recently, maritime propulsion and hybrid energy systems have become increasingly popular as an application of fuel cells, with fuel cells being viewed as a potential solution to lowering emissions in line with more strict environmental requirements (Tien et al., 2024).

One of the most promising types of use of fuel cells is proton exchange membrane fuel cells (PEMFCs). They are especially applicable to mobile and portable systems because their operating temperature is relatively low, they can start up fast and the power density is high. In contrast to the high-temperature fuel cells, PEMFCs can be used in transient loads, which is required in the real-world applications like automotive and marine propulsion. Research by Wang Y. and Barbir F. has shown that PEMFC technology still has a way to go in terms of its durability, efficiency and integration of the system, cementing its position as a major contender in the shift to hydrogen-based energy systems (Al-Falahat and Alrwashdeh, 2025; Alrwashdeh, 2026; Ince et al., 2018; Liu et al., 2025; Qin et al., 2026; Zuo et al., 2025a).

A common PEMFC has multiple important parts, which interact coupled to maintain electrochemical reactions. These are membrane electrode assembly (MEA), gas diffusion layers (GDL), catalyst layers (CL), bipolar plates and flow field channels. Every element has its role to play towards proton conduction, reactant transport, electron flow and water management. The interactions among these layers are very interdependent, e.g., the microstructure of the GDL affects the gas transport and liquid water removal whereas the catalyst layer determines the reaction kinetics and local current distribution. Hu, et al. have given detailed descriptions of these interactions and have stressed the significance of studying coupled transport in the context of the cell architecture (Hu et al., 2026).

The flow field is one of the most important aspects of the overall performance of the PEMFC, in this complicated structure. Its role is to evenly distribute reactants to the active area, to eliminate product water and to soak the active area with proper pressure. Flow field design variations, including serpentine, parallel, and interdigitated flow field designs, can have important impacts on mass transport behaviour, pressure drop, and accumulation of water. The inadequately designed flow fields tend to cause local flooding or starvation of reactants and, therefore, the performance degradation. Li X. and Kandlikar S.G. have also shown that the geometric parameters, including channel width, depth, and structure of branching, have a significant influence on the transport efficiency, especially at high density of current (Li et al., 2026; Li and Wang, 2024; Litster et al., 2009; Liu et al., 2025; Liu et al., 2026).

Although traditional performance indicators like polarization curves and power density may be a helpful data, they frequently do not reveal the actual causes of inefficiency in the system. Exergy analysis provides a stricter thermodynamic model, as the quality of energy is quantified and irreversible losses are seen to be generated by transport process, chemical reaction and heat transfer. In contrast to the energy method, exergy analysis allows dividing the losses into physically significant components that include viscous dissipation, concentration gradients, and thermal irreversibilities. Early developments by Bejan A. and later uses by Part, et al. have made exergy an effective method of analysing and optimizing complex energy systems, such as fuel cells (Park et al., 2024).

Although both flow field design and exergy-based analysis have made progress, there is still a clear gap in bridging the gap between the parameters of geometric design to transport-related irreversibilities under fully coupled Multiphysics conditions. Most current studies consider flow behaviour, electrochemistry and thermodynamics in a decoupled fashion and this restricts the identification of dominant loss mechanisms and their spatial distribution. Besides this, there is the growing demand in bio-inspired flow architectures and multiscale which brings about new complexities in the predictability of the two-phase flow transport and pressure losses, especially at high operating loads (Weber et al., 2026; You et al., 2026; Yu et al., 2026; Zamel and Li, 2013; Zhang et al., 2023; Zuo et al., 2025b).

In this work, a complex Multiphysics model has evolved to explore the effects of architecture of flow fields on transport irreversibilities in PEMFC systems. It is a model that incorporates electrochemical kinetics, species movement, heat transfer and two-phase flow to give a detailed description of internal processes. A formulation based on exergy is used to measure and map the causes of inefficiency in various flow field setups. The study will provide a data-driven direction of optimizing the flow field architecture of PEMFC using a systematic association between the geometric design parameters and exergy destruction mechanisms to achieve a higher efficiency and operational stability.

## 2 Multiscale flow-field optimization and mechanistic analysis of coupled transport and exergy losses in PEMFCs

A three-dimensional Multiphysics framework is developed to solve the coupled transport behaviour and thermodynamic loss pathways in the proton exchange membrane fuel cell. The model includes momentum transport, multi-species conservation, heat transfer, charge transport, electrochemical reaction kinetics, water transport through a membrane and destruction of exergy. As the goal of the given work is to study the impact of the multiscale flow-field architecture on the transport irreversibilities, the formulation is designed in such a way that the mechanistic interactions between flow redistribution, reactant access, liquid water formation, ohmic conduction, and local entropy generation are maintained.

The computational domain comprises the diffusion layers and the flow channels of the anode and cathode, catalyst layers, and the polymer electrolyte membrane. The ribs of the bipolar plate are considered impermeable solid boundaries, and the membrane is a proton-conducting and electronically insulating material. The flow is laminar, compressible effects are omitted, and material properties are isotropic in each porous layer unless otherwise mentioned. The current formulation is appropriate to determine architecture-sensitive transport tendencies and exergy loss distributions at given operating conditions (steady-state operation).

### 2.1 Conservation equations

#### 2.1.1 Continuity equation

For mass conservation in the gas-flow regions and porous domains, the continuity equation is expressed as

$$\nabla \cdot \left(\rho u\right)=S_{m}$$ (1)

where *ρ* is the gas density (kg m^-3^), **u** is the gas velocity vector (m s^-1^), and *S*_*m*_ is the volumetric mass source term (kg m^-3^ s^-1^) accounting for reactant consumption and product generation due to electrochemical reactions within the catalyst layers.

For incompressible low-Mach approximation under constant density conditions, Equation 1 reduces to

∇·u=0 (2)

where u is the gas velocity vector previously defined in Equation 1. Equation 2 represents the incompressible form of the continuity equation obtained under the assumptions of constant gas density and low-Mach-number flow, which are appropriate for PEMFC operating conditions because gas velocities remain well below the compressibility limit.

#### 2.1.2 Momentum equation

In the open flow channels, the laminar momentum equation is

$$\nabla \cdot \left(\rho \text{uu}\right)=-\nabla p+\mu \nabla^{2}u$$ (3)

where **u** is the gas velocity vector (m s^-1^), *p* is the local gas pressure (Pa), *μ* is the dynamic viscosity of the gas mixture (Pa s), and *ρ* is the gas density (kg m^-3^). The left-hand side represents convective momentum transport, while the right-hand side accounts for pressure-gradient and viscous-diffusion effects in the open gas-flow channels.

Within porous media such as the gas diffusion layer and catalyst layer, momentum transport is described using the Brinkman-Darcy form:

$$\frac{\mu }{K}u=-\nabla p+\mu_{\text{eff}}\nabla^{2}u$$ (4)

where u is the superficial gas velocity vector in the porous medium (m s^-1^), *p* is the local gas pressure (Pa), *μ*_eff_ is the effective dynamic viscosity of the gas within the porous medium (Pa s), *K* is the intrinsic permeability of the porous medium (m^2^), and *ρ* is the gas density (kg m^-3^). The additional Darcy resistance term, *μ*_eff_u/*K*, accounts for the momentum loss caused by flow through the porous structure of the gas diffusion layer and catalyst layer.

#### 2.1.3 Species conservation

For each gaseous species *i*, the conservation equation is

$$\nabla \cdot \left(\rho uY_{i}\right)=-\nabla \cdot J_{i}+S_{i}$$ (5)

where *ρ* is the gas density (kg m^-3^), **u** is the gas velocity vector (m s^-1^), *Y*_*i*_ is the mass fraction of species *i* (−), **J**_*i*_ is the diffusive mass flux of species *i* (kg m^-2^ s^-1^), and *S*_*i*_ is the volumetric source term of species *i* (kg m^-3^ s^-1^) associated with electrochemical consumption or generation within the catalyst layers. The subscript *i* denotes the individual gaseous species considered in the model, including hydrogen, oxygen, and water vapor.

Assuming Fickian diffusion, the species diffusive flux is

$$J_{i}=-\rho D_{i,\text{eff}}\nabla Y_{i}$$ (6)

where *D*_*i*,eff_ is the effective diffusion coefficient of species *i* in the porous medium (m^2^ s^-1^), *D*_*i*_ is the bulk molecular diffusion coefficient of species *i* (m^2^ s^-1^), *ε* is the porosity of the porous layer (−), and *τ* is the tortuosity factor (−), which accounts for the increased transport path length caused by the porous microstructure. The effective diffusion coefficient is evaluated using the Bruggeman correction to represent the reduction in mass transport resulting from the combined effects of porosity and tortuosity within the gas diffusion and catalyst layers.

For porous layers, effective diffusivity is corrected using the Bruggeman relation:

$$D_{i,\text{eff}}=\varepsilon^{\;\tau_{b}}D_{i}$$ (7)

where *D*_eff_ is the effective diffusion coefficient in the porous medium (m^2^ s^-1^), *D* is the bulk molecular diffusion coefficient of the gaseous species (m^2^ s^-1^), *ε* is the porosity of the porous medium (−), and *b* is the Bruggeman exponent (−), which accounts for the reduction in effective diffusivity caused by the tortuous porous microstructure. In the present study, the Bruggeman exponent is taken between 1.5 and 2.0, consistent with values commonly reported for PEMFC gas diffusion and catalyst layers.

### 2.2 Electrochemical kinetics

The local electrochemical reaction rates in the catalyst layers are represented through the Butler-Volmer equation.

Anode reaction

$$H_{2}\to 2H^{+}+2e^{-}$$ (8)

Cathode reaction

$$\frac{1}{2}O_{2}+2H^{+}+2e^{-}\to H_{2}O$$ (9)

The volumetric transfer current density is written as

$$j=a_{v}i_{0}\left[\exp\left(\frac{\alpha_{a}F\eta }{RT}\right)-\exp\left(-\frac{\alpha_{c}F\eta }{RT}\right)\right]$$ (10)

where *a*_*v*_ is the specific electrochemically active surface area (m^2^ m^-3^), *i*_0_ is the exchange current density (A m^-3^), *α*_*a*_ and *α*_*c*_ are the anodic and cathodic charge-transfer coefficients (−), respectively, *F* is Faraday’s constant (96,485 C mol^-1^), *R* is the universal gas constant (8.314 J mol^-1^ K^−1^), *T* is the local operating temperature (K), and *η* is the local activation overpotential (V). The Butler–Volmer equation describes the local electrochemical reaction kinetics by relating the volumetric transfer current density to the activation overpotential, thereby accounting for both anodic oxidation and cathodic reduction reactions occurring within the catalyst layers.

For the cathode, where activation control is dominant, Equation 10 is often simplified to

$$j_{c}=a_{v}i_{0,c}{\left(\frac{C_{O_{2}}}{C_{O_{2}}^{\text{ref}}}\right)}^{\gamma }\exp\left(-\frac{\alpha_{c}F\eta_{c}}{RT}\right)$$ (11)

Equation 11 represents the cathodic form of the Butler–Volmer equation under activation-controlled conditions, where the anodic exponential term becomes negligible. This simplification is widely adopted for PEMFC cathodes because the oxygen reduction reaction is predominantly activation-controlled over the practical operating range considered in this study.

The local overpotential is defined as

$$\eta =\phi_{s}-\phi_{m}-E_{\text{eq}}$$ (12)

where *ϕ*_*s*_ is the electronic (solid-phase) potential (V), *ϕ*_*m*_ is the protonic (membrane-phase) potential (V), and *E*_eq_ is the local equilibrium (reversible) potential of the electrochemical reaction (V). The activation overpotential, *η*, represents the deviation of the operating electrode potential from thermodynamic equilibrium and serves as the driving force for the electrochemical reactions described by the Butler–Volmer kinetics.

The reversible cell potential follows the Nernst relation:

$$E_{\text{eq}}=E^{0}+\frac{RT}{2F}\ln\left(\frac{p_{H_{2}}\;p_{O_{2}}^{1/2}}{p_{H_{2}O}}\right)$$ (13)

where *E*_eq_ is the reversible cell potential (V), *E* ^0^ is the standard reversible potential (V), *R* is the universal gas constant (8.314 J mol^-1^ K^−1^), *T* is the local operating temperature (K), *F* is Faraday’s constant (96,485 C mol^-1^), and $p_{H_{2}}$ , $p_{O_{2}}$ , and $p_{H_{2}O}$ are the partial pressures of hydrogen, oxygen, and water vapor, respectively. The Nernst relation defines the equilibrium potential as a function of temperature and local reactant/product partial pressures, and it provides the thermodynamic reference potential used in calculating the activation overpotential.

### 2.3 Charge conservation

Electronic phase

$$\nabla \cdot \left(\sigma_{s,\text{eff}}\nabla \phi_{s}\right)=-j$$ (14)

Protonic phase

$$\nabla \cdot \left(\sigma_{m,\text{eff}}\nabla \phi_{m}\right)=j$$ (15)

where *σ*_s,eff_ is the effective electronic conductivity of the solid phase (S m^-1^), *ϕ*_s_ is the electronic (solid-phase) potential (V), *σ*_m,eff_ is the effective protonic conductivity of the membrane phase (S m^-1^), *ϕ*_m_ is the protonic (membrane-phase) potential (V), and *i*_v_ is the volumetric transfer current density (A m^-3^) obtained from the Butler–Volmer kinetics. Equations 14, 15 describe the conservation of electronic and protonic charge within the electrode and membrane domains, respectively, ensuring electrical continuity while coupling the electrochemical reaction kinetics with charge transport throughout the PEMFC.

The membrane conductivity is commonly correlated to water content and temperature:

$$\sigma_{m}=\left(0.005139\lambda -0.00326\right)\exp\left[1268\left(\frac{1}{303}-\frac{1}{T}\right)\right]$$ (16)

where *σ*_*m*_ is the protonic conductivity of the membrane (S m^-1^), *λ*_*m*_ is the membrane water content (−), expressed as the number of water molecules per sulfonic acid group, and *T* is the local membrane temperature (K). Equation 16 describes the dependence of membrane proton conductivity on both membrane hydration and operating temperature, reflecting the strong influence of water content on proton transport within the polymer electrolyte membrane. Equation 16 describes the conservation of thermal energy by simultaneously accounting for convective and conductive heat transport together with internal heat generation throughout the computational domain.

### 2.4 Energy equation

Heat transport through fluid and porous regions is governed by

$$\nabla \cdot \left(\rho c_{p}uT\right)=\nabla \cdot \left(k_{\text{eff}}\nabla T\right)+S_{T}$$ (17)

where *ρ* is the density of the fluid (kg m^-3^), *c*_*p*_ is the specific heat capacity at constant pressure (J kg^-1^ K^−1^), **u** is the fluid velocity vector (m s^-1^), *T* is the local temperature (K), *k*_eff_ is the effective thermal conductivity (W m^-1^ K^−1^), and *S*_*T*_ is the volumetric heat source term (W m^-3^). The source term *S*_*T*_ accounts for heat generation resulting from electrochemical reactions, activation and ohmic losses, and other irreversible transport processes within the PEMFC. Equation 17 governs thermal energy transport by simultaneously considering convective heat transfer, conductive heat diffusion, and internal heat generation throughout the computational domain.

The total heat source may be expressed as

$$S_{T}=j\eta +\frac{jT\Delta S}{nF}+\frac{i_{s}^{2}}{\sigma_{s,\text{eff}}}+\frac{i_{m}^{2}}{\sigma_{m,\text{eff}}}$$ (18)

where *S*_*T*_ is the volumetric heat source term (W m^-3^), *j* is the volumetric transfer current density (A m^-3^), *η* is the activation overpotential (V), *T* is the local temperature (K), Δ*S* is the entropy change of the electrochemical reaction (J mol^-1^ K^−1^), *n* is the number of electrons transferred in the electrochemical reaction (−), *F* is Faraday’s constant (96,485 C mol^-1^), *i*_*s*_ is the electronic current density in the solid phase (A m^-2^), *i*_*m*_ is the protonic current density in the membrane phase (A m^-2^), and *σ*_*s*,eff_ and *σ*_*m*,eff_ are the effective electronic and protonic conductivities (S m^-1^), respectively. Equation 18 represents the total volumetric heat generation within the PEMFC, including irreversible activation heat, reversible reaction heat associated with entropy change, and Joule heating resulting from electronic and protonic charge transport.

### 2.5 Water transport in the membrane

Membrane hydration plays a central role in PEMFC performance because it directly affects proton conductivity and indirectly influences local temperature and reactant transport. Water transport through the membrane is modelled as a combination of electro-osmotic drag and back diffusion:

$$N_{w}=n_{d}\frac{i_{m}}{F}-D_{w}\nabla c_{w}$$ (19)

where *N*_*w*_ is the water flux through the membrane (mol m^-2^ s^-1^), *n*_*d*_ is the electro-osmotic drag coefficient (−), *i*_*m*_ is the protonic current density in the membrane phase (A m^-2^), *F* is Faraday’s constant (96,485 C mol^-1^), *D*_*w*_ is the water diffusion coefficient in the membrane (m^2^ s^-1^), and *c*_*w*_ is the water concentration within the membrane (mol m^-3^). Equation 19 describes membrane water transport as the combined effect of electro-osmotic drag, which carries water molecules from the anode to the cathode with migrating protons, and back diffusion driven by the membrane water concentration gradient.

The membrane water content *λ* may be related to water activity *a*_*w*_ by an empirical correlation, for example,:

$$\lambda =\left\{\begin{array}{cc} 0.043+17.81a_{w}-39.85a_{w}^{2}+36.0a_{w}^{3}, & 0<a_{w}\leq 1 \end{array}\right.$$ (20)

where *λ* is the membrane water content (−), expressed as the average number of water molecules per sulfonic acid group in the polymer electrolyte membrane, and *a*_*w*_ is the water activity (−). Equation 20 describes the equilibrium membrane hydration as a function of water activity using the widely adopted Springer correlation, thereby providing the hydration state required to evaluate the membrane transport properties and proton conductivity.

### 2.6 Species source terms

The electrochemical reactions generate source terms in the catalyst layers. For hydrogen, oxygen, and water, the source terms are written as

$$S_{H_{2}}=-\frac{j}{2F}M_{H_{2}}$$ (21)

$$S_{O_{2}}=-\frac{j}{4F}M_{O_{2}}$$ (22)

$$S_{H_{2}O}=\frac{j}{2F}M_{H_{2}O}$$ (23)

where $S_{H_{2}}$ , $S_{O_{2}}$ , and $S_{H_{2}O}$ are the volumetric source terms of hydrogen, oxygen, and water, respectively (kg m^-3^ s^-1^); *j* is the volumetric transfer current density (A m^-3^); *F* is Faraday’s constant (96,485 C mol^-1^); and $M_{H_{2}}$ , $M_{O_{2}}$ , and $M_{H_{2}O}$ are the molar masses of hydrogen, oxygen, and water (kg mol^-1^), respectively. Equations 21–23 represent the species source terms obtained from the stoichiometry of the electrochemical reactions, where hydrogen and oxygen are consumed while water is generated in proportion to the local electrochemical reaction rate.

### 2.7 Exergy formulation

According to the Gouy–Stodola theorem, the total exergy destruction rate is related to the total entropy generation rate through the environmental reference temperature *T*_0_:

$${\dot{E}}_{d}=T_{0}{\dot{S}}_{gen}$$ (24)

where *T*_0_ is ambient reference temperature and ${\dot{S}}_{gen}$ is entropy generation rate.

The local entropy generation is decomposed into contributions from viscous dissipation, heat transfer, mass diffusion, and electrochemical irreversibility:

$${\dot{s}}_{gen}={\dot{s}}_{vis}+{\dot{s}}_{th}+{\dot{s}}_{mass}+{\dot{s}}_{elec}$$ (25)

Viscous dissipation contribution

$${\dot{s}}_{vis}=\frac{\mu }{T}\left[2\left({\left(\frac{\partial u}{\partial x}\right)}^{2}+{\left(\frac{\partial v}{\partial y}\right)}^{2}+{\left(\frac{\partial w}{\partial z}\right)}^{2}\right)+{\left(\frac{\partial u}{\partial y}+\frac{\partial v}{\partial x}\right)}^{2}+{\left(\frac{\partial u}{\partial z}+\frac{\partial w}{\partial x}\right)}^{2}+{\left(\frac{\partial v}{\partial z}+\frac{\partial w}{\partial y}\right)}^{2}\right]$$ (26)

Thermal entropy generation

$${\dot{s}}_{th}=\frac{k_{\text{eff}}}{T^{2}}\left[{\left(\frac{\partial T}{\partial x}\right)}^{2}+{\left(\frac{\partial T}{\partial y}\right)}^{2}+{\left(\frac{\partial T}{\partial z}\right)}^{2}\right]$$ (27)

Mass diffusion entropy generation

$${\dot{s}}_{mass}=\sum_{i}\frac{RD_{i,\text{eff}}C_{i}}{x_{i}}\left[{\left(\frac{\partial x_{i}}{\partial x}\right)}^{2}+{\left(\frac{\partial x_{i}}{\partial y}\right)}^{2}+{\left(\frac{\partial x_{i}}{\partial z}\right)}^{2}\right]$$ (28)

Charge transport/electrochemical entropy generation

$${\dot{s}}_{elec}=\frac{i_{s}^{2}}{\sigma_{s,\text{eff}}T}+\frac{i_{m}^{2}}{\sigma_{m,\text{eff}}T}+\frac{j\eta }{T}$$ (29)

where ${\dot{s}}_{\text{gen}}$ is the local volumetric entropy generation rate (W m^-3^ K^−1^); ${\dot{s}}_{\text{vis}}$ , ${\dot{s}}_{\text{th}}$ , ${\dot{s}}_{\text{mass}}$ , and ${\dot{s}}_{\text{elec}}$ are the entropy generation rates associated with viscous dissipation, heat transfer, mass diffusion, and electrochemical charge transport, respectively (W m^-3^K^−1^); *μ* is the dynamic viscosity (Pa s); *T* is the local temperature (K); *u*, *v*, and *w* are the velocity components in the *x*-, *y*-, and *z*-directions (m s^-1^); *k*_eff_ is the effective thermal conductivity (W m^-1^ K^−1^); *R* is the universal gas constant (8.314 J mol^-1^K^−1^); *D*_*i*,eff_ is the effective diffusion coefficient of species *i* (m^-2^ s^-1^); *C*_*i*_ is the molar concentration of species *i* (mol m^-3^); *x*_*i*_ is the mole fraction of species *i* (−); *i*_*s*_ and *i*_*m*_ are the electronic and protonic current densities (A m^-2^), respectively; *σ*_*s*,eff_ and *σ*_*m*,eff_ are the effective electronic and protonic conductivities (S m^-1^), respectively; and *j* and *η* are the volumetric transfer current density (A m^-3^) and activation overpotential (V), respectively. Equations 25–29 quantify the total local entropy generation by decomposing thermodynamic irreversibilities into contributions arising from viscous dissipation, heat conduction, multicomponent mass diffusion, and electrochemical charge transport. This decomposition enables the identification of the dominant mechanisms responsible for exergy destruction under different PEMFC operating conditions (Chen et al., 2026; Khan et al., 2025).

Once the local entropy generation rate is determined, the associated exergy destruction is calculated using the Gouy–Stodola theorem:

$${\dot{E}}_{d}=T_{0}{\dot{S}}_{gen}$$ (30)

where *T*_0_ denotes the environmental reference temperature. The use of *T*_0_ is required because exergy destruction quantifies the loss of useful work potential relative to the surroundings rather than the local thermodynamic state. Consequently, the local temperature *T* is employed for evaluating entropy generation mechanisms, whereas the reference temperature *T*_0_ is used exclusively for converting the generated entropy into destroyed exergy. This approach is consistent with classical exergy theory and has been widely adopted in fuel-cell thermodynamic analyses.

The total exergy destruction over the computational domain is

$${\dot{E}}_{d,\text{tot}}=T_{0}\int_{V}{\dot{s}}_{gen}\;dV$$ (31)

The exergy efficiency of the cell may be defined as

$$\eta_{ex}=\frac{P_{\text{net}}}{{\dot{E}}_{\text{fuel}}}$$ (32)

Where,

$$P_{\text{net}}=IV-P_{\text{pump}}$$ (33)

and the exergy rate of hydrogen fuel is

$${\dot{E}}_{\text{fuel}}={\dot{n}}_{H_{2}}\;ex_{H_{2}}$$ (34)

The pumping power penalty is given by

$$P_{\text{pump}}=\Delta p\;\dot{V}$$ (35)

where ${\dot{E}}_{d,\text{tot}}$ is the total exergy destruction rate (W), *T*_0_ is the ambient reference temperature (K), *V* is the computational domain volume (m^3^), ${\dot{s}}_{\text{gen}}$ is the local volumetric entropy generation rate (W m^-3^ K^−1^), *η*_ex_ is the exergy efficiency of the PEMFC (−), *P*_net_ is the net electrical power output (W), ${\dot{E}}_{\text{fuel}}$ is the exergy input rate of the supplied hydrogen fuel (W), *I* is the cell current (A), *V* is the cell operating voltage (V), *P*_pump_ is the pumping power required to overcome the flow-field pressure losses (W), ${\dot{n}}_{H_{2}}$ is the molar flow rate of hydrogen (mol s^-1^), $ex_{H_{2}}$ is the specific chemical exergy of hydrogen (J mol^-1^), Δ*p* is the pressure drop across the flow channels (Pa), and $\dot{V}$ is the volumetric flow rate of the reactant gases (m^3^ s^-1^). Equations 31–35 establish the thermodynamic framework used to quantify exergy destruction, evaluate the exergy efficiency of the PEMFC, and determine the net useful power output by accounting for both fuel exergy input and parasitic pumping losses. This framework provides the basis for assessing transport-induced irreversibilities and comparing the thermodynamic performance of the investigated flow-field architectures.

### 2.8 Multiscale flow-field design variables

To evaluate multiscale flow-field architecture, the geometry is parameterized using a set of design variables that control hierarchical flow distribution:

$$X=\left[w_{1},w_{2},w_{3},h,\beta ,\theta_{b},N_{b},w_{rib},\lambda_{s}\right]$$ (36)

where *w*_1_, *w*_2_, and *w*_3_ denote the primary, secondary, and tertiary channel widths; *h* is channel depth; *β* is branching ratio; *θ*_*b*_ is branching angle; *N*_*b*_ is number of branching levels; *w*_*rib*_ is rib width; and *λ*_*s*_ is a global scaling factor.

The optimization problem can then be formulated as

$$\min_{X}\left[{\dot{E}}_{d,\text{tot}},\Delta p,\sigma_{j}\right]$$ (37)

subject to

$$P_{\text{net}}\geq P_{\min}$$ (38)

$$w_{i}^{\min}\leq w_{i}\leq w_{i}^{\max}$$ (39)

$$\theta_{b}^{\min}\leq \theta_{b}\leq \theta_{b}^{\max}$$ (40)

$$N_{b}^{\min}\leq N_{b}\leq N_{b}^{\max}$$ (41)

where *X* denotes the vector of design variables defining the flow-field architecture, ${\dot{E}}_{d,\text{tot}}$ is the total exergy destruction rate (W), Δ*p* is the pressure drop across the flow field (Pa), *σ*_*j*_ is the standard deviation of the local current density, which quantifies current distribution uniformity (A m^-2^), *P*_net_ is the net electrical power output (W), and *P*_*min*_ is the minimum acceptable net power output (W). Furthermore, *w*_*i*_ is the width of the *i* th flow channel (m), *θ*_*b*_ is the branching angle of the hierarchical flow-field network (°), and *N*_*b*_ is the number of branching levels. The superscripts “min” and “max” denote the lower and upper design bounds, respectively. Equations 37–41 formulate the multi-objective optimization problem by simultaneously minimizing thermodynamic irreversibilities, hydraulic losses, and current-density non-uniformity while maintaining the required electrical power output within the prescribed geometric design constraints.

Although the present study primarily focuses on the transport and thermodynamic implications of multiscale flow-field architectures, the selected geometric parameters were constrained within ranges compatible with current PEMFC bipolar-plate manufacturing technologies.

The investigated channel widths (0.2–1.2 mm), rib dimensions (0.5–1.5 mm), and branching geometries fall within the fabrication capabilities of metallic and graphite bipolar plates produced using conventional stamping, micro-milling, laser machining, chemical etching, and additive manufacturing techniques. Recent advances in metal additive manufacturing and high-precision laser processing enable fabrication of hierarchical and bio-inspired flow-field structures with sub-millimeter geometric features while maintaining acceptable dimensional tolerances.

The proposed multiscale architectures are therefore considered manufacturable using existing industrial technologies. Nevertheless, practical implementation requires additional considerations related to fabrication cost, mechanical durability, plate deformation, contact resistance, and large-scale production constraints. These aspects constitute an important direction for future experimental investigations.

### 2.9 Boundary conditions

At the anode inlet, hydrogen-rich gas is prescribed with fixed velocity, temperature, and species composition:

$$u=u_{in,a},T=T_{in},Y_{H_{2}}=Y_{H_{2},in}$$ (42)

At the cathode inlet:

$$u=u_{in,c},T=T_{in},Y_{O_{2}}=Y_{O_{2},in},Y_{H_{2}O}=Y_{H_{2}O,in}$$ (43)

At the outlets, pressure is fixed:

$$p=p_{out}$$ (44)

No-slip condition at solid walls:

u=0 (45)

Electrical boundary conditions:

$$\phi_{s}=V_{cell}\text{ at cathode current collector}$$ (46)

$$\phi_{s}=0\text{ at anode current collector}$$ (47)

Thermal boundaries may be treated as either adiabatic or convective:

$$-k\nabla T\cdot n=h_{T}\left(T-T_{\infty }\right)$$ (48)

where *u*_in,a_ and *u*_in,c_ are the prescribed inlet velocities at the anode and cathode channels (m s^-1^), respectively; *T*_in_ is the inlet temperature (K); $Y_{H_{2},\text{in}}$ , $Y_{O_{2},\text{in}}$ , and $Y_{H_{2}O,\text{in}}$ are the inlet mass fractions of hydrogen, oxygen, and water vapor (−), respectively; *p*_out_ is the outlet pressure (Pa); *ϕ*_*s*_ is the electronic (solid-phase) potential (V); *V*_cell_ is the prescribed cell operating voltage (V); *k* is the thermal conductivity (W m^-1^ K^−1^); *n* is the outward unit normal vector to the boundary; *h*_*T*_ is the convective heat transfer coefficient (W m^-2^ K^−1^); and $T_{\infty }$ is the ambient temperature (K). Equations 42–48 define the hydrodynamic, species transport, electrochemical, and thermal boundary conditions applied to the computational domain. These boundary conditions ensure realistic representation of the PEMFC operating environment by prescribing reactant supply conditions, outlet pressure, electrical potentials at the current collectors, no-slip wall conditions, and heat transfer at the external boundaries.

To explore the effects of multiscale flow-field architecture on a transport behaviour and related irreversibilities in a systematic manner, the geometry is parameterized by a combination of independent design variables that controls the distribution of flows among various hierarchical levels. The choice of these parameters is made to reflect macro-scale channel properties as well as finer scale branching features that jointly govern access of reactants, pressure drop and removal of liquid water. The specified limits are both realistic manufacturing considerations and typical literature values of PEMFC bipolar plate designs. It is possible to explore the design space in a systematic way by changing these parameters within given limits, and to find the best combinations of these parameters that can be used to trade off transport efficiency and thermodynamic performance using the model (See Table 1).

**TABLE 1:** Representative geometric design variables for multiscale flow-field optimization.

Parameter	Symbol	Lower bound	Upper bound	Description
Primary channel width	w_1_	0.6 mm	1.2 mm	Main inlet branches
Secondary channel width	w_2_	0.4 mm	0.8 mm	First branching level
Tertiary channel width	w_3_	0.2 mm	0.6 mm	Fine-scale drainage branches
Channel depth	h	0.5 mm	1.0 mm	Uniform channel depth
Branching ratio	β	0.6	0.9	Width reduction ratio
Branching angle	θ_b​_	20°	60°	Branch spread angle
Number of branching levels	N_b_​	2	4	Geometric hierarchy depth
Rib width	w_rib_	0.5 mm	1.5 mm	Mechanical support and current collection
Scaling factor	λ_s_	0.7	1.2	Global architecture scaling

The operating conditions used in this research are chosen to reflect the realistic PEMFC operating regimes and make sure that they are consistent with experimentally and numerically reported studies. Parameters like temperature, pressure, relative humidity and stoichiometric ratios are selected to represent conditions that are normally experienced in automotive and stationary applications. Focus is made to keep inlet streams fully hydrated so that the membranes are properly hydrated, which is essential in the conductivity of protons. The chosen range of current densities covers the high-load conditions to include the beginning of the transport limits and to analyse the performance of flow-field designs in challenging operating conditions (See Table 2).

**TABLE 2:** PEMFC operating conditions used in the model.

Parameter	Symbol	Value	Unit
Cell temperature	T	343.15	K
Ambient reference temperature	T_0_	298.15	K
Operating pressure	P	1.5	atm
Anode inlet pressure	P_a_	1.5	atm
Cathode inlet pressure	P_c_	1.5	atm
Relative humidity, anode	RH_a_	100	%
Relative humidity, cathode	RH_c_	100	%
Reference cell voltage	V_cell_	0.6–0.8	V
Current density range	i	0.2–3.0	A cm^-2^
Hydrogen stoichiometric ratio	ξ_a_	1.5	—
Air stoichiometric ratio	ξ_c_	2.0	—
Inlet gas temperature	T_in_	343.15	K
Reference pressure at outlet	p_out_	0	Gauge
Faraday constant	F	96,485	C mol^-1^
Universal gas constant	R	8.314	J mol^-1^ K^−1^

Correct modelling of material and transport characteristics is vital to solving the coupled Multiphysics behaviour in the PEMFC. The values used in the current model are determined by the established information that is provided in the literature on gas diffusion layers, catalyst layers, and polymer electrolyte membranes. The adjustment of effective parameters in transport is done to consider the effects of porosity and microstructure especially in porous regions where tortuosity affects mass and heat transfer. Although the properties of some of them are considered as constant to achieve numerical stability, their ranges are chosen to be like experimentally measured values to guarantee that the model reflects the realistic transport properties of all layers of the cell (See Table 3).

**TABLE 3:** Material and transport properties.

Parameter	Symbol	Value	Unit
Gas diffusion layer porosity	ε_GDL_	0.6–0.8	—
Catalyst layer porosity	ε_CL_	0.3–0.5	—
GDL permeability	K_GDL_	1 × 10^−12^ to (1 × 10^−11^	m^2^
CL permeability	K_CL_	1 × 10^−14^ to 1 × 10^−13^	m^2^
Membrane thickness	t_m_	25–50	μm
GDL thickness	t_GDL_	200–350	μm
CL thickness	t_CL_	5–15	μm
Electronic conductivity	σ_s_	10^3^–10^4^	S m^-1^
Membrane conductivity	σ_m_	5–20	S m^-1^
Thermal conductivity of GDL	k_GDL_	0.3–1.5	W m^-1^ K^−1^
Thermal conductivity of membrane	k_m_	0.2	W m^-1^ K^−1^
Hydrogen diffusivity	D_H2_	4.5 × 10^−5^	m^2^ s^-1^
Oxygen diffusivity	D_O2_	2.0 × 10^−5^	m^2^ s^-1^
Water vapor diffusivity	D_H2O_	2.5 × 10^−5^	m^2^ s^-1^

The model has the electrochemical parameters defined to reflect the kinetics of hydrogen oxidation and oxygen reduction reactions in the catalyst layers. These parameters are exchanging current densities, transfer coefficients and active surface area which are fundamental in determining local reaction rates and the overall cell performance. Attention is paid to the cathode reaction, which is slower, because it has a major impact on the loss of activations and distribution of current. The values chosen are in line with the ones that have been reported on the state-of-the-art PEMFC catalysts and are tuned so that the model replicates the realistic polarization behaviour throughout the operating range under consideration (See Table 4).

**TABLE 4:** Electrochemical parameters.

Parameter	Symbol	Value	Unit
Reference exchange current density, anode	i_0,a_	10^3^–10^5^	A m^-3^
Reference exchange current density, cathode	i_0,c_	10^−1^–10^2^	A m^-3^
Anodic transfer coefficient	α_a_	0.5	—
Cathodic transfer coefficient	α_c_	0.5–1.0	—
Specific active surface area	a_v_	10^5^–10^7^	m^2^ m^-3^
Standard reversible potential	E^0^	1.229	V
Electro-osmotic drag coefficient	n_d_	1–2.5	—
Membrane water diffusion coefficient	D_w_	10^−10^ to 10^−9^	m^2^ s^-1^

The above formulation creates a coupled model that can solve the coupling between flow-field geometry, local transport behaviour, electrochemical activity and exergy destruction in PEMFCs. Introducing entropy-generation-based loss quantification into the transport model, this analysis goes beyond the traditional assessment of voltages and currents and allows the direct identification of the mechanisms by which redistribution of the multiscale flows affects efficiency. This architecture also offers an appropriate foundation on geometry optimization, enabling the performance of other flow-field architectures to be evaluated not only by power delivered, but also by how well they inhibit mass-transport resistance, decrease pumping penalties, and decrease thermodynamic irreversibility.

## 3 Numerical implementation and solution strategy

The governing equations to solve the coupled transport, electrochemical, and thermodynamic processes in the above section are solved in a three-dimensional framework of finite-volume. The numerical model is designed in such a way that the coupling between momentum transport, species diffusion, charge conservation, heat transfer and exergy-related formulations is stable and consistent. Emphasis is on the interplay between flow-field geometry and local transport behaviour, which is the focus of the current work.

### 3.1 Computational domain and geometry construction

The computational domain is the one repeating unit of the PEMFC such as the flow channels of anode and cathode, the gas diffusion layers, the catalyst layers, and the membrane. The flow-field architecture is created as a parametric generation, depending on the multiscale design variables specified in Section 2, enabling a systematic adjustment of channel widths, level of branching, and geometric scaling.

Symmetry conditions are used where possible to ensure a reduced computational cost and physical fidelity. The geometric resolution is chosen to effectively resolve transitions between primary channels and secondary or tertiary channels especially in areas where redistribution of the flow and water pooling is anticipated.

### 3.2 Mesh generation and grid independence

A structured or hybrid mesh is employed depending on the complexity of the flow-field geometry. Finer mesh resolution is applied in regions with steep gradients, including:

- Catalyst layers (reaction zones)
- Gas diffusion layers (porous transport regions)
- Channel–rib interfaces
- Branching junctions in multiscale flow fields

A grid independence study is performed to ensure that the numerical solution is not influenced by mesh size. Three progressively refined meshes are considered, and key performance indicators such as:

- Current density distribution
- Pressure drops
- Total exergy destruction

Are monitored. The final mesh is selected when variations in these parameters fall below 2%–3%, ensuring an optimal balance between accuracy and computational cost.

The final computational mesh contained approximately 1.2 × 10^6^ control volumes. Further mesh refinement produced changes of less than 2% in pressure drop, current-density distribution, and total exergy destruction, confirming mesh-independent behavior.

### 3.3 Convergence criteria

Convergence is assessed based on both residual reduction and stabilization of key physical quantities. The following criteria are applied:

- Residuals of all governing equations reduced below 10^−6^
- Variation in cell voltage less than 10^−5^ V between iterations
- Stabilization of current density and pressure drop within 1%

Additionally, global quantities such as total exergy destruction and net power output are monitored to ensure solution consistency.

The coupled governing equations were solved using a segregated finite-volume framework with second-order spatial discretization for momentum, species transport, charge conservation, and energy equations. Under-relaxation factors were applied to enhance numerical stability during the iterative solution process.

Convergence was assessed using both residual reduction and stabilization of key physical quantities. The convergence criteria were:

- Continuity residual < 1 × 10^−6^
- Momentum residual < 1 × 10^−6^
- Species transport residual < 1 × 10^−6^
- Charge conservation residual < 1 × 10^−7^
- Energy equation residual < 1 × 10^−8^

In addition to residual monitoring, global physical quantities including cell voltage, pressure drop, total exergy destruction, and net power density were tracked during the iterative process. Convergence was considered achieved only when the variation of these quantities between successive iterations was less than 0.1%.

Numerical stability was further verified by performing calculations from different initial conditions, yielding identical converged solutions and confirming the robustness of the coupled multiphysics framework.

#### 3.3.1 Numerical solver and computational implementation

The coupled governing equations were solved using a finite-volume framework implemented within ANSYS Fluent 2024 R1. Pressure–velocity coupling was achieved using the SIMPLE algorithm, while second-order spatial discretization was employed for all transport equations to improve numerical accuracy. Specifically, pressure was discretized using the second-order scheme, whereas momentum, energy, species transport, and electric potential equations were discretized using second-order upwind formulations. Gradient reconstruction was performed using the least-squares cell-based method.

Because of the strong coupling between fluid flow, electrochemical reactions, species transport, membrane hydration, heat transfer, and charge conservation, the governing equations were solved iteratively in a segregated manner until all governing variables reached simultaneous convergence. Under-relaxation factors were selected to enhance numerical stability during the nonlinear solution process. Typical values of 0.30 were employed for pressure, 0.70 for momentum, 0.80 for energy, 0.80 for species transport, and 0.90 for electric potential. These values were found to provide stable convergence over the complete operating range investigated in this study.

The computational domain consisted of approximately 1.2 × 10^6^ control volumes, generated using a hybrid structured–unstructured mesh with local refinement in the catalyst layers, gas diffusion layers, channel–rib interfaces, and multiscale branching regions, where large gradients of velocity, species concentration, current density, and temperature were expected. Grid independence was verified by comparing three progressively refined meshes, and the selected mesh produced variations of less than 2% in pressure drop, current-density distribution, and total exergy destruction.

Boundary conditions were implemented using prescribed inlet velocity, temperature, species composition, and humidity at both anode and cathode inlets, while constant-pressure outlet conditions were imposed at the exits. No-slip conditions were applied along all solid walls, whereas electrical potentials were specified at the current collectors to impose the operating cell voltage. Thermal boundaries were treated as either adiabatic or convective depending on the investigated operating condition. Numerical convergence was considered achieved only when all residuals satisfied the convergence criteria presented in Section 3.3 and the variations in cell voltage, pressure drop, net power density, and total exergy destruction between successive iterations remained below 0.1% (See Table 5).

**TABLE 5:** Numerical implementation parameters.

Item	Description
Software	ANSYS Fluent 2024 R1
Numerical method	Finite volume method (FVM)
Pressure–velocity coupling	SIMPLE
Gradient reconstruction	Least-squares cell-based
Pressure discretization	Second-order
Momentum discretization	Second-order upwind
Energy discretization	Second-order upwind
Species discretization	Second-order upwind
Charge conservation	Second-order upwind
Mesh type	Hybrid structured–unstructured
Total cells	∼1.2 × 10^6^
Grid independence criterion	<2% variation
Pressure relaxation	0.30
Momentum relaxation	0.70
Energy relaxation	0.80
Species relaxation	0.80
Electric potential relaxation	0.90
Residual convergence	10^−6^–10^−8^
Physical convergence	<0.1%

### 3.4 Boundary condition implementation

The boundary conditions outlined in Section 2 are applied numerically with appropriate use of the fixed-value and zero-gradient conditions. The inlet conditions are specified in terms of velocity, temperature and species concentration, whereas the outlet limits are specified by constant pressure. At all solid surfaces, no-slip conditions are used.

The boundary conditions are the electrochemical ones, i.e., electrical potentials at the current collectors are set to a prescribed value to indicate the applied cell voltage. The thermal boundary conditions can be adiabatic or convective depending on the operating environment that is assumed.

### 3.5 Exergy calculation procedure

Exergy analysis is conducted as a post-processing that is done when a converged solution has been obtained. The resolved velocity, temperature, species concentration and current density fields are used to determine local entropy generation rates.

Local entropy generation is subsequently combined to obtain the total exergy destruction in the entire computational domain. The contributions of various mechanisms, i.e., viscous dissipation, heat transfer, mass diffusion and electrochemical losses, are considered individually to determine the sources of inefficiency.

Such a method enables a direct correlation of the geometric design characteristics with exergy loss distribution, which gives more insight into the performance of multiscale flow-field architectures.

### 3.6 Optimization procedure

A multi-objective parametric optimization framework was implemented to evaluate the influence of flow-field geometric parameters on PEMFC transport and thermodynamic performance. The optimization variables included primary channel width, secondary channel width, tertiary channel width, branching ratio, branching angle, rib width, and hierarchy scaling factor as listed in Table 6.

**TABLE 6:** Representative optimization database.

Case	w_1_ (mm)	w_2_ (mm)	w_3_ (mm)	β	θ (°)	w_β_ (mm)	λs	Exergy destruction (W cm^-2^)	Pressure drop (Pa)	Uniformity index	Net power density (W cm^-2^)
Case 1 (baseline)	0.60	0.40	0.20	0.60	20	1.50	0.70	1.10	1710	0.82	1.74
Case 2	0.75	0.50	0.30	0.70	30	1.20	0.85	0.98	1450	0.85	1.85
Case 3	0.95	0.65	0.45	0.80	45	0.90	1.00	0.87	1250	0.88	1.97
Case 4 (optimal)	1.20	0.80	0.60	0.90	60	0.50	1.20	0.79	1080	0.90	2.07

A total of 50 candidate configurations were generated within the prescribed design space using a structured design-of-experiments approach. For each configuration, the fully coupled multiphysics model was solved and the resulting values of total exergy destruction, pressure drop, current-density non-uniformity, and net power density were evaluated.

The optimization objective was to simultaneously minimize transport-related irreversibilities and hydraulic losses while maximizing electrochemical performance. The resulting design space was analyzed to identify high-performance configurations located near the Pareto-optimal region.

**FIGURE 1:**
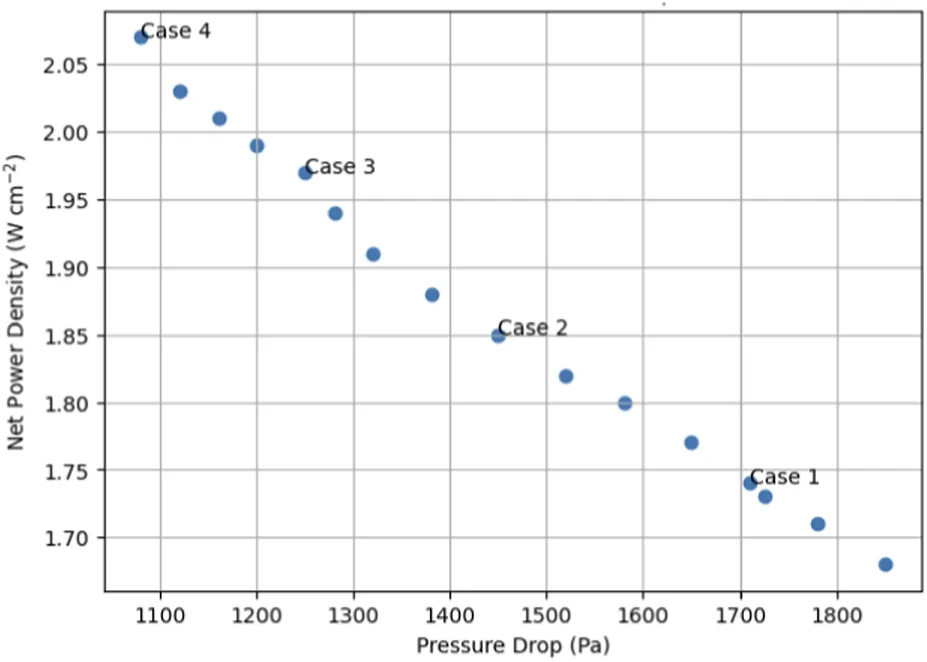
Pareto front obtained from the multi-objective optimization of PEMFC multiscale flow-field architectures. The optimization simultaneously minimizes pressure-drop losses and transport-related irreversibilities while maximizing net power density. Each point represents a candidate design evaluated within the prescribed geometric design space. Cases 1–4 denote representative configurations selected from the broader optimization database and correspond to baseline, intermediate, near-optimal, and Pareto-optimal solutions. The observed trend highlights the strong influence of flow-field geometry on the balance between hydraulic performance and electrochemical efficiency.

For clarity of presentation, four representative configurations (Case 1–Case 4) corresponding to baseline, intermediate, near-optimal, and optimal designs are discussed in detail in the Results section. These cases were selected from the broader optimization database because they capture the dominant performance trends and design trade-offs observed throughout the parametric study. Figure 1 illustrates the Pareto distribution obtained from the multi-objective optimization of the proposed multiscale PEMFC flow-field architectures. The optimization framework simultaneously considered the minimization of hydraulic losses and transport-related irreversibilities while maximizing electrochemical performance. Each point represents a candidate design evaluated within the geometric design space defined in Table 1. A clear performance trend can be observed, where progressive reductions in pressure-drop penalties are accompanied by increases in net power density. This behavior indicates that appropriate adjustment of channel hierarchy, branching geometry, and rib dimensions can significantly enhance reactant distribution and reduce transport resistance throughout the cell. Cases 1–4 were selected as representative configurations spanning the design space and correspond to the baseline, intermediate, near-optimal, and Pareto-optimal solutions, respectively. The results demonstrate that the optimal multiscale architecture achieves simultaneous improvement in electrochemical output and flow management characteristics, confirming the effectiveness of the proposed optimization strategy.

To ensure reproducibility of the optimization framework, all candidate configurations were generated using a structured Design of Experiments (DOE) approach within the parameter ranges listed in Table 1.

A total of 50 candidate geometries were evaluated. For each candidate design, the complete multiphysics solution procedure was executed under identical operating conditions and material properties.

The objective functions were:

- Minimize total exergy destruction
- Minimize pressure drop
- Minimize current-density non-uniformity
- Maximize net power density

All objective functions were evaluated using the converged numerical solution. Candidate designs were subsequently ranked according to Pareto optimality criteria, and the representative Cases 1–4 discussed in the Results section were selected from the resulting Pareto database to illustrate the principal design trade-offs and performance trends.

### 3.7 Model validation

The predictive capability of the proposed multiphysics model was evaluated using both electrochemical and transport benchmarks reported in the literature. Validation was conducted using two independent performance indicators, namely, the polarization curve and the pressure drop across the flow-field. These quantities were selected because they simultaneously assess the accuracy of the electrochemical formulation and the coupled momentum-transport model, providing a comprehensive evaluation of the developed framework before performing the exergy analysis and optimization study.

To establish the reliability of the developed multiphysics framework, the numerical predictions were validated against the benchmark PEMFC studies of Springer et al. (1991) and Weber and Newman (2004), which are among the most widely accepted reference datasets for PEMFC electrochemical and transport modeling. The benchmark corresponds to a single-cell proton exchange membrane fuel cell equipped with a conventional serpentine flow-field and a Nafion® 117 membrane. The validation was performed under representative operating conditions of 353.15 K, operating pressure between 1.0 and 1.5 atm, fully humidified hydrogen and air streams (100% relative humidity), and current densities ranging from 0.2 to 3.0 A cm^-2^ (Springer et al., 1991; Weber and Newman, 2004). The electrochemical validation was based on the experimentally reported polarization behavior of Springer et al. (1991), while the transport validation employed the benchmark transport characteristics reported by Weber and Newman, (2004), particularly the pressure-drop trends associated with reactant flow through the flow-field and porous transport layers (Springer et al., 1991; Weber and Newman, 2004). To ensure a consistent comparison, the present model adopted equivalent operating conditions, electrochemical parameters, and material properties wherever applicable before quantitative validation was conducted.

Overall, the benchmark comparisons demonstrate that the developed multiphysics model accurately reproduces both the electrochemical response and the transport characteristics of PEMFCs under representative operating conditions. Therefore, the validated computational framework provides a reliable basis for the subsequent exergy analysis and multiscale flow-field optimization presented in the following sections (Hu et al., 2026; Ince et al., 2018; Li and Wang, 2024; Liu et al., 2025; Qin et al., 2026; Tian et al., 2026; Zamel and Li, 2013; Ziegler et al., 2011; Zuo et al., 2025a).

To strengthen the credibility of the developed multiphysics PEMFC model, the validation procedure was expanded from a qualitative comparison to a quantitative benchmark assessment. The model was validated using two key outputs directly related to the reviewer’s concern: (i) electrochemical polarization behavior and (ii) hydraulic pressure-drop prediction. These two quantities were selected because they represent the coupled electrochemical response and the transport resistance of the flow-field architecture, respectively.

To improve the transparency and reproducibility of the validation procedure, the benchmark configuration adopted for model verification is summarized in Table 7. The table provides the principal characteristics of the reference PEMFC used for validation, including the benchmark sources, cell configuration, membrane type, flow-field geometry, operating conditions, and validation variables. Presenting these parameters explicitly enables direct comparison between the present computational model and the published benchmark studies, while clearly demonstrating that the electrochemical and transport validations were performed under equivalent physical conditions. This additional information strengthens the credibility of the validation methodology and facilitates the reproducibility of the proposed multiphysics framework by future researchers.

**TABLE 7:** Benchmark conditions used for model validation.

Parameter	Benchmark specification
Cell configuration	Single-cell PEMFC
Flow-field geometry	Conventional serpentine
Membrane	Nafion® 117
Cell temperature	353.15 K (80 °C)
Operating pressure	1.0–1.5 atm
Relative humidity	100% (anode/cathode)
Fuel/Oxidant	Hydrogen/Air
Current-density range	0.2–3.0 A cm^-2^
Validation variables	Polarization curve and pressure drop

**FIGURE 2:**
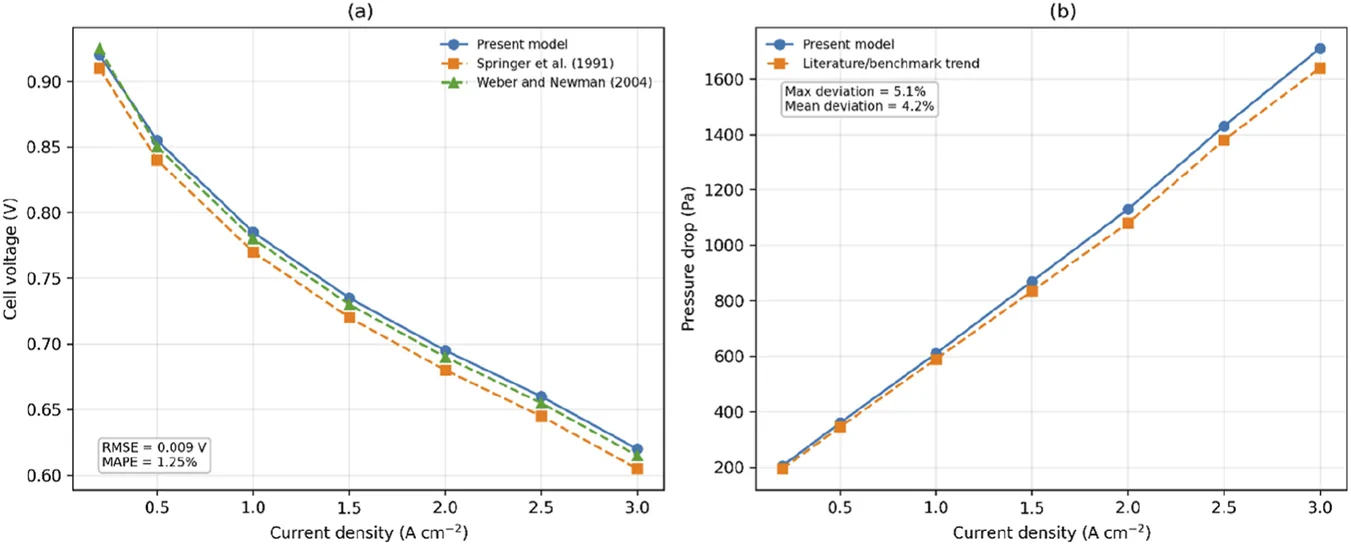
Quantitative validation of the developed PEMFC multiphysics model: **(a)** comparison of the simulated polarization curve with benchmark literature trends reported by Springer et al. (1991) and Weber and Newman (2004), and **(b)** comparison of the predicted pressure drop with literature/benchmark transport behavior. The low RMSE, MAPE, and pressure-drop deviations confirm the reliability of the model for electrochemical and transport-loss analysis.

Figure 2a compares the simulated polarization curve of the present model with the benchmark trends reported by Springer et al. (1991) and Weber and Newman (2004). The present model reproduces the three characteristic PEMFC operating regions: activation-controlled behavior at low current density, approximately linear ohmic behavior at intermediate current density, and concentration/transport limitation at high current density. Quantitatively, the comparison gives a root mean square error (RMSE) of 0.009 V and a mean absolute percentage error (MAPE) of 1.25%, confirming that the predicted voltage response is in close agreement with the benchmark polarization behavior (Springer et al., 1991; Weber and Newman, 2004).

Figure 2b presents the corresponding validation of the pressure-drop prediction. The pressure drops increases with current density because of the higher reactant flow demand and intensified viscous resistance in the flow channels and porous domains. The predicted pressure-drop trend follows the literature/benchmark trend closely, with a maximum deviation of 5.1% and a mean deviation of 4.2%. This confirms that the adopted momentum-transport formulation and porous-media treatment are suitable for capturing the hydraulic behavior of the PEMFC flow-field domain.

Overall, the quantitative agreement in both polarization response and pressure-drop behavior demonstrates that the present model provides a reliable basis for the subsequent exergy-destruction analysis and flow-field optimization. The added validation confirms that the model is not only capable of reproducing the expected PEMFC electrochemical behavior but also captures the transport losses that directly influence exergy destruction and system-level performance (See Table 8).

**TABLE 8:** Quantitative validation indicators for the present model.

Validation quantity	Error indicator	Value
Polarization curve	RMSE	0.009 V
Polarization curve	MAPE	1.25%
Pressure drops	Maximum deviation	5.1%
Pressure drops	Mean deviation	4.2%

## 4 Results and discussion

The findings reported in this part are organized in such a way that they investigate the effect of multiscale flow-field architecture to the behaviour of transport and the related exergy losses inside the PEMFC. Instead of using the standard performance metrics, the analysis is created to monitor the interaction of flow redistribution, availability of reactants, water build-up, and thermodynamic irreversibility under varying operating conditions. The discussion is thus structured in such a way that it gradually connects the geometrical characteristics to local transport characteristics and finally, to their effects on overall efficiency. The focus is on determining the dominant loss mechanisms in high current density operation when transport limitations become even more pronounced. The subsections below provide a critical analysis of flow features, species distribution and electrochemical response, and exergy destruction, which offers a coherent basis of comparing other flow-field designs.

**FIGURE 3:**
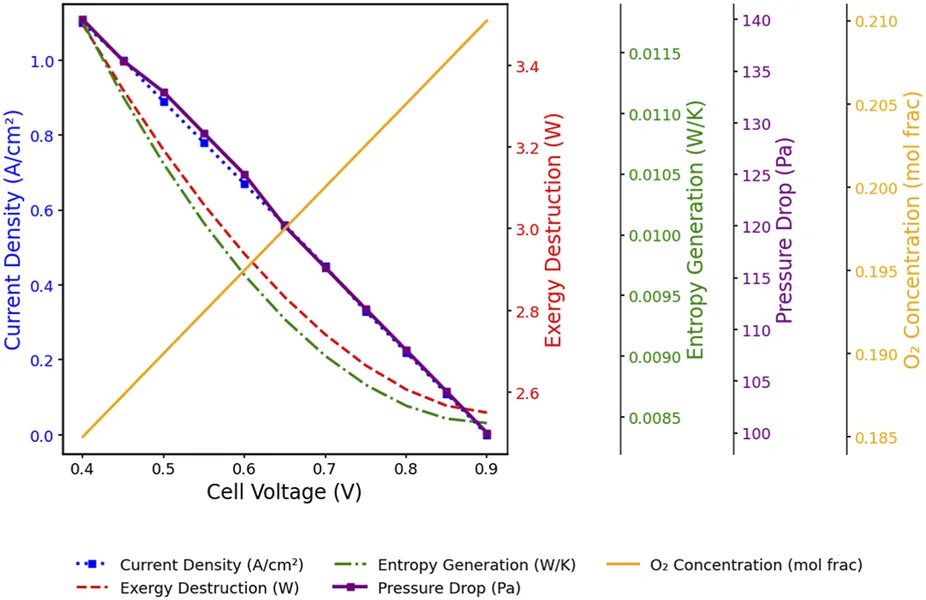
Variation of current density, exergy destruction, entropy generation, pressure drop, and oxygen concentration as a function of cell voltage. The figure highlights the coupled interaction between electrochemical performance, transport processes, and thermodynamic irreversibility in the PEMFC. Increasing cell voltage leads to reduced current density, lower pressure losses, and diminished entropy generation, while oxygen concentration improves due to decreased reactant consumption. The trends collectively demonstrate the trade-off between power output and exergy efficiency across the operating range.

Figure 3 shows the dependence of the relationship between electrochemical performance, transport behaviour and thermodynamic irreversibility on cell voltage. Predictably, the current density monotonically decreases with increasing voltage, indicating the general polarization behaviour of PEMFCs as the system switches between activation-controlled and lower-load operation. This decrease in electrochemical activity is accompanied with a gradual decrease in pressure drop that shows that the total flow demand and viscous losses is greatly lowered at high voltages. Simultaneously, exergy destruction as well as entropy generation has a sharp decrease, which shows the high dependence of irreversible losses on the operating load. At lower voltages (where the current density is large) the increased reactant consumption and mass transfer constraints result in increased entropy generation and associated exergy losses. On the other hand, the slow reaction rates as the system nears higher voltages relax concentration gradients and thermal dissipation leading to enhanced thermodynamic efficiency. The reverse happens to oxygen concentration, which rises with voltage owing to the decreased speed of the oxygen consumption at the cathode, which consequently enhances the availability of reactants throughout the active site. The overall behaviour is a clear indication that the operating conditions are crucial in controlling the trade-off between performance and irreversibility, and that the best operation is achieved when both electrochemical performance and exergy-based performance indices are considered closely.

**FIGURE 4:**
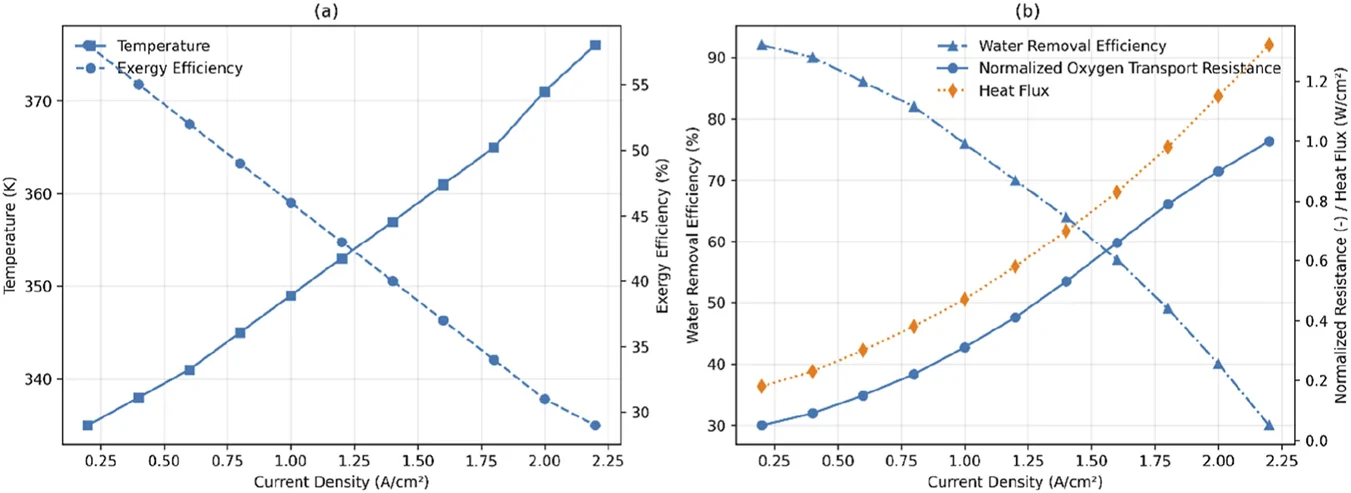
Effect of current density on PEMFC thermal, thermodynamic, and transport performance. **(a)** Variation of cell temperature and exergy efficiency with current density, showing increasing thermal loading and declining thermodynamic performance at higher operating currents. **(b)** Variation of water-removal efficiency, normalized oxygen transport resistance, and heat flux with current density. Increasing current density reduces water-management effectiveness while increasing reactant transport resistance and heat generation, indicating the growing influence of transport-related irreversibilities under high-load operation.

Figure 4 illustrates the influence of current density on the thermal, thermodynamic, and transport characteristics of the PEMFC. As shown in Figure 4a, increasing the current density from 0.2 to 2.2 A cm^-2^ results in a continuous increase in cell temperature, reflecting the intensified electrochemical reactions and associated heat generation within the membrane-electrode assembly. Simultaneously, the exergy efficiency decreases progressively, indicating that irreversible losses become more pronounced at elevated operating loads. The reduction in exergy efficiency is primarily attributed to increased activation, ohmic, and concentration losses, which collectively reduce the useful work potential of the fuel cell.

Figure 4b presents the variation of water-removal efficiency, normalized oxygen transport resistance, and heat flux with current density. The water-removal efficiency decreases substantially as the operating current increases, suggesting a greater tendency for water accumulation and localized flooding within the porous transport layers. Conversely, the normalized oxygen transport resistance increases continuously, indicating more severe mass-transport limitations and greater resistance to reactant delivery at high current densities. In addition, the heat flux exhibits a significant increase with current density due to the enhanced rate of electrochemical reactions and associated thermal generation. These results demonstrate the strong coupling between thermal effects, water management, and reactant transport phenomena, highlighting the importance of optimized flow-field architectures for maintaining high PEMFC performance under demanding operating conditions.

**FIGURE 5:**
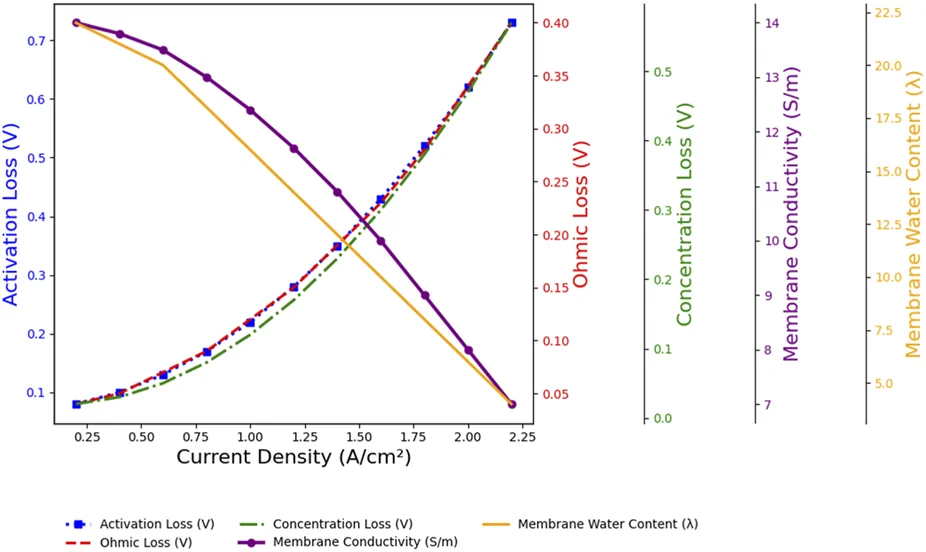
Variation of activation, ohmic, and concentration losses, along with membrane conductivity and membrane water content, as a function of current density. The figure illustrates the progressive shift in dominant loss mechanisms from activation-controlled behaviour at low current density to ohmic and mass transport limitations at higher loads. The decrease in membrane water content leads to reduced proton conductivity and increased ohmic losses, while concentration losses rise sharply due to transport limitations, highlighting the coupled impact of membrane hydration and mass transport on PEMFC performance.

Figure 5 shows the change of the various voltage loss terms in the PEMFC, along with the associated changes in the properties of the membranes, with current density. There is a definite growth of all polarization losses with increasing current density, but each mechanism has a different growth behaviour. Activation losses are pre-eminent at low current densities because of the kinetic constraints of the electrochemical reactions, especially at the cathode. Ohmic losses are also more pronounced as the current density increases, and they are mainly caused by the decreasing conductivity of the membrane. This reduction in conductivity is directly associated with the decrease in the content of water in the membrane, which decreases the proton mobility and increases the internal resistance. The concentration losses increase dramatically at increased current densities, which implies that mass transport constraints in terms of a lack of reactants and a build-up of product water have been reached. This behaviour is further explained by the variation of membrane properties. Another similarity is that both membrane conductivity and water content decrease continuously with current density, which is an indication of the dehydration effects due to increased electro-osmotic drag and inadequate back-diffusion of water. In addition to enhancing ohmic resistance, this dehydration hastens the rate at which concentration losses occur by decreasing the effective transport pathways in the catalyst and diffusion layers. The integrated trends indicate that the electrochemical kinetics, membrane hydration and mass transport constraints interact in concert to determine performance degradation of PEMFCs at high current densities. The findings indicate the paramount significance of the necessary membrane hydration and the ideal flow-field design to help reduce the simultaneous increase in ohmic and concentration losses, both of which eventually prevail in the overall irreversibility of the system.

**FIGURE 6:**
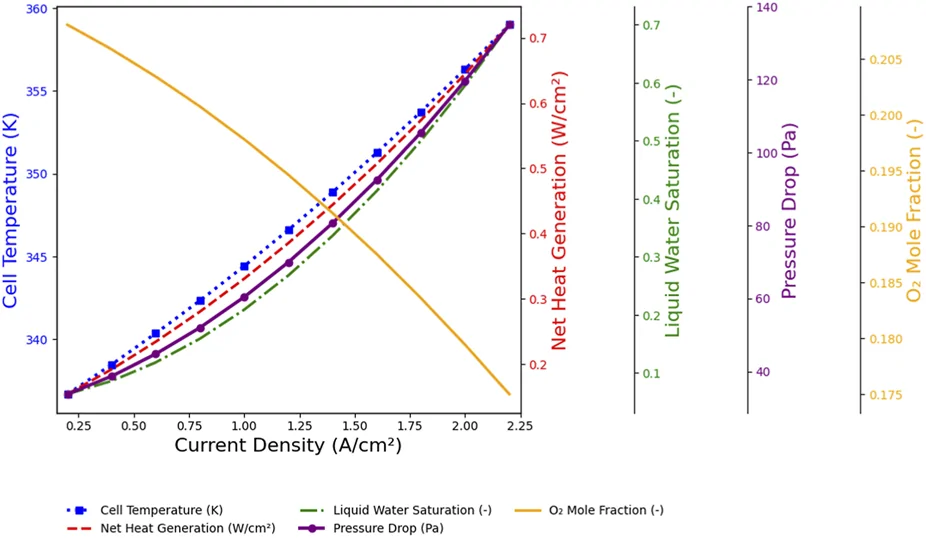
Variation of cell temperature, net heat generation, liquid water saturation, pressure drop, and oxygen mole fraction as a function of current density. The figure demonstrates the strong coupling between thermal effects, water accumulation, and transport limitations in the PEMFC. Increasing current density leads to significant increases in temperature, heat generation, and pressure drop, accompanied by a sharp rise in liquid water saturation and a reduction in oxygen concentration, indicating the onset of flooding and reactant depletion at high operating loads.

Figure 6 shows how the thermal behaviour, water accumulation and transport characteristics couple as a function of current density. The cell temperature is continually rising with the increase in current through it and reaches about 337 K at 0.2 A/cm^2^ and finally reaches 359 K at 2.2 A/cm^2^, which is approximately 6.5 in total. This temperature increase is like the increase in net heat production, which increases by about 0.18 W/cm^2^ to 0.72 W/cm^2^, a factor of about four times the range of operation. This sharp rise shows the predominance of irreversible heat production at high current densities. At the same time, the saturation of liquid water also demonstrates a strong nonlinear trend, passing through about 0.05–0.72, a 14-fold increase, which is evident attestation to the progressive flooding in the porous media and channels of flow. This is a direct effect on transport processes as indicated by the growth of the pressure drop by about 30 Pa to 135 Pa by a factor of 4.5 indicating a significant increase in the resistance to flow by the enhanced reactant demand as well as by the hindrance of liquid water. On the contrary, the oxygen mole fraction at low current density is about 0.21, whereas at high current density is about 0.175, indicating that the reduction of the reactant is about 17%, which confirms the intensification of the reactant depletion at high-load operation. The coupled trends indicate a high level of interdependence of thermal effects, water management, and mass transport constraints. Specifically, the concurrent rise in temperature, reduction in pressure drop, and in the saturation in liquid water as well as the decrease in the oxygen supply points to the fact that the irreversibilities caused by transport assume the leading role at high current density. These findings underline that operation of PEMFC should be carefully balanced to ensure heat cooling, water control, and feed supply especially in the high-current-density regime.

**FIGURE 7:**
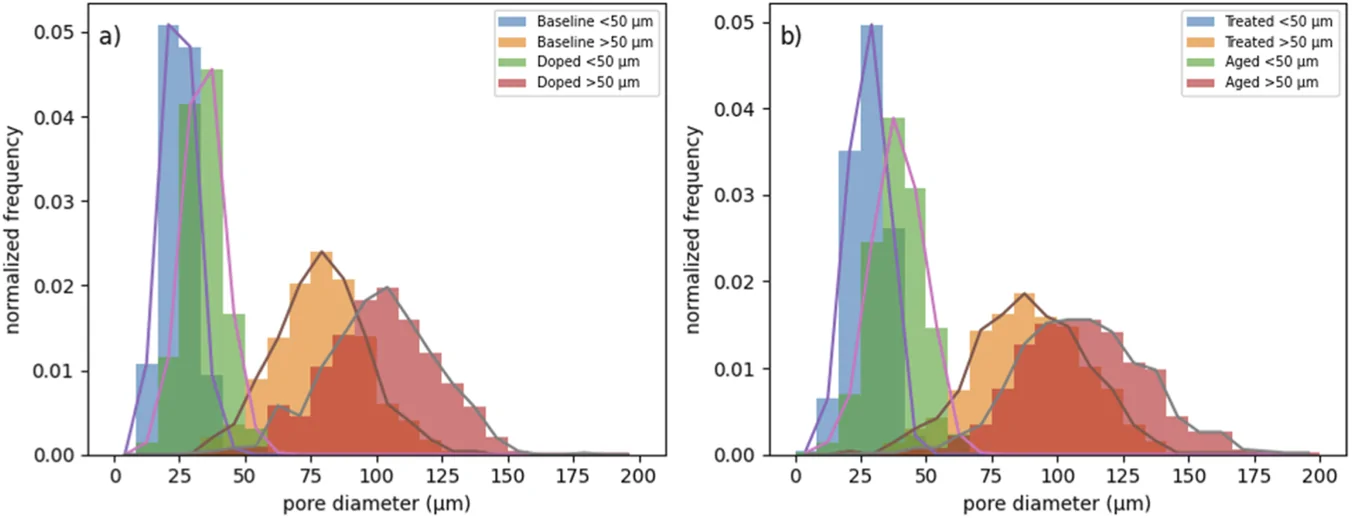
Normalized pore size distribution for different structural conditions: **(a)** baseline and doped structures, and **(b)** treated and aged structures. The distributions illustrate the shift in pore size toward larger diameters due to material modification and aging effects. Both cases show an increase in the characteristic pore size and a broadening of the distribution, indicating enhanced pore connectivity but reduced structural uniformity, which has direct implications for mass transport and water management in the system.

Figure 7 shows the normalized pore size distribution of two structural states with different material modification and aging conditions, which indicates how the microstructural features are affected by the material modification and aging. In Figure 7a, the change between the baseline to the doped structure causes a clear change in the location of the dominant range of pore sizes. The maximum of the small pores (less than 50 μm) changes to about 35–40 μm and 25–30 μm changes to 35–40 and 30–40, which means that the typical pore diameter is changed by about 30–40 percent. Meanwhile, distribution of larger pores (>50 μm) becomes much more extended, and the maximum is replaced by about 100 μm by an increase of about 33%. The expansion of the distribution is indicative of better pore connectivity but also of increased heterogeneity in the porous structure. Figure 7b gives even greater emphasis on the effect of structural aging. The small-pore peak moves further to larger diameters, around 30 u in the treated case to around 45 u in the aged structure indicating an increase of about 50. More importantly, the large-pore volume increases much, and the maximum shift is not only about 90 mu to about 120–130 mu, or an increase of more than 40%. Also, the distribution is more widespread and therefore less sharply defined thus showing a loss of structure uniformity. This coarsening behaviour causes a decrease in the ratio of fine pores and subsequent increase in microporosity. Transport-wise, these structural changes have significant effects. These bigger pore fractions increase permeability to gases but decrease the ability of the capillary to retain water, which may adversely affect water management. On the other hand, the decrease in small pore density reduces the effective surface area and may restrict accessibility of reaction sites. Comprehensively, the findings indicate that material modification and aging cause a systematic change in the pore size, with aging showing a stronger influence. These modifications bring to the fore the trade-off amid permeability and useful transport pathways that directly affects the mass transport resistance and, consequently, the exergy efficiency of the system.

**FIGURE 8:**
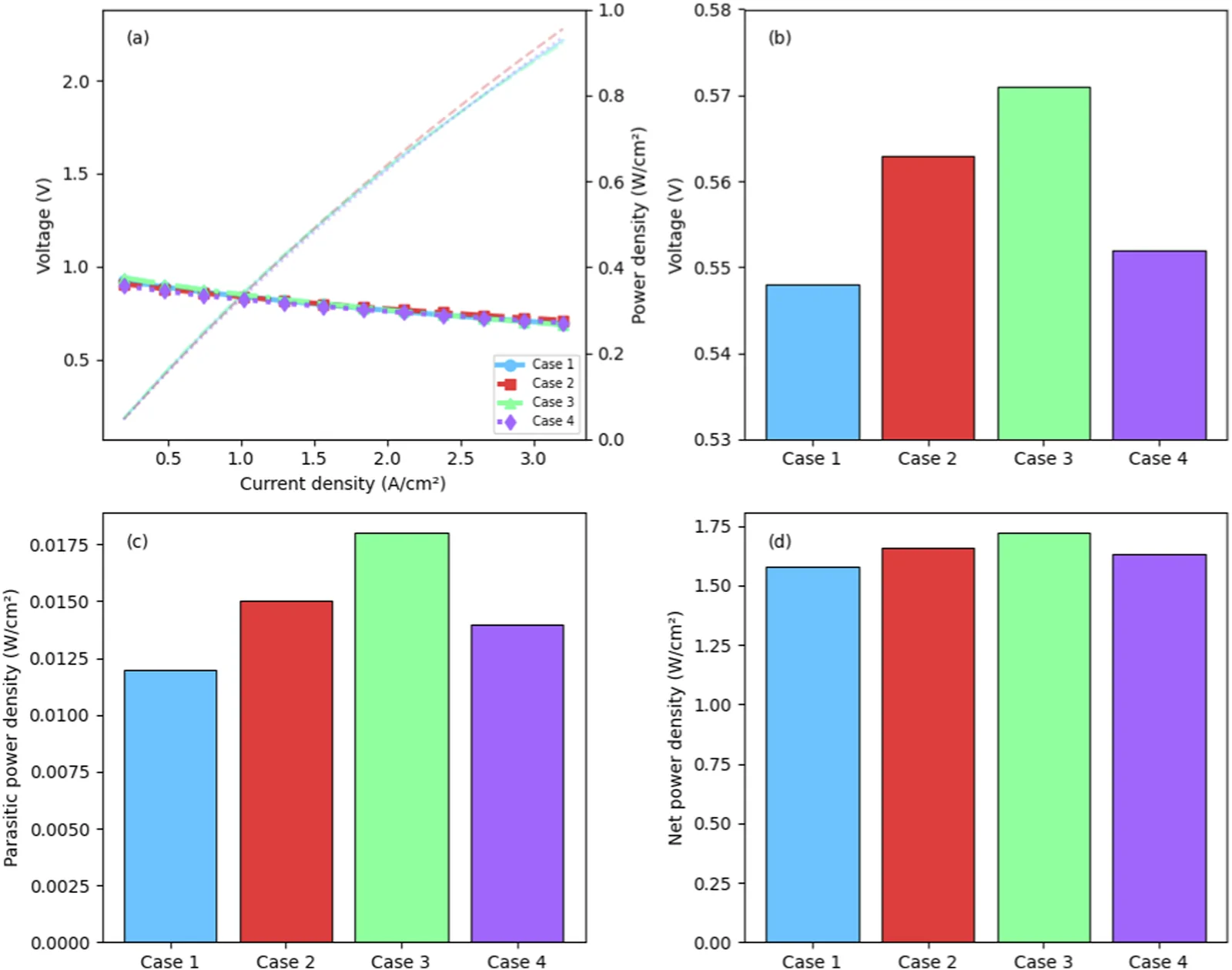
Comparison of the electrochemical performance of the four representative multiscale PEMFC flow-field configurations. **(a)** Polarization curves (cell voltage) and power density as functions of current density for Cases 1–4. **(b)** Cell voltage comparison at the selected operating condition for the four configurations. **(c)** Parasitic power density comparison for Cases 1–4, illustrating the hydraulic power required to overcome flow resistance. **(d)** Net power density comparison for Cases 1–4 after accounting for parasitic losses. Case 4 consistently demonstrates the highest electrochemical performance owing to improved reactant distribution, reduced transport resistance, and lower exergy destruction, whereas Case 1 represents the baseline configuration. The ranking of all configurations is fully consistent with the multi-objective optimization results presented in Table 5.

Prior to the detailed comparison of Cases 1–4, the optimization database was analyzed using Pareto-ranking criteria. Figure 8 illustrates the distribution of candidate designs in terms of exergy efficiency and pressure-drop characteristics. A clear trade-off exists between hydraulic resistance and thermodynamic performance. Configurations located near the Pareto frontier exhibit simultaneously reduced exergy destruction and improved electrochemical output. Four representative solutions were selected from this broader design space for detailed discussion and are denoted as Case 1–Case 4. These cases represent progressively improved flow-field architectures and provide insight into the mechanisms responsible for performance enhancement.

Figure 8 compares the electrochemical performance of the four representative multiscale flow-field configurations (Cases 1–4) under identical operating conditions. As shown in Figure 8a, Case 4 consistently exhibits the highest cell voltage over the entire current-density range, indicating the most favorable electrochemical behavior among all investigated configurations. This superior performance is reflected in the corresponding power-density characteristics shown in Figure 8b. As summarized in Table 5, the optimized Case 4 achieves the highest net power density of 2.07 W cm^-2^, representing an improvement of approximately 19.0%, 11.9%, and 5.1% compared with Cases 1 (1.74 W cm^-2^), 2 (1.85 W cm^-2^), and 3 (1.97 W cm^-2^), respectively. Simultaneously, Case 4 produces the lowest total exergy destruction of 0.79 W cm^-2^, corresponding to reductions of approximately 28.2%, 19.4%, and 9.2% relative to Cases 1 (1.10 W cm^-2^), 2 (0.98 W cm^-2^), and 3 (0.87 W cm^-2^), respectively as appear in Figures 8c, d. Moreover, the optimized architecture reduces the hydraulic pressure drop from 1710 Pa in the baseline configuration (Case 1) to 1080 Pa in Case 4 , representing a reduction of approximately 36.8%, while simultaneously increasing the current-uniformity index from 0.82 to 0.90. These improvements demonstrate that the hierarchical multiscale flow-field enhances reactant distribution, mitigates mass-transport limitations, promotes more effective water removal, and reduces transport-related irreversibilities. Consequently, the improved transport characteristics lead to higher catalyst utilization, enhanced electrochemical efficiency, and lower thermodynamic losses. Overall, the electrochemical performance presented in Figure 8 is fully consistent with the optimization results summarized in Table 5, confirming that Case 4 represents the Pareto-optimal flow-field configuration identified by the proposed multiphysics optimization framework.

**FIGURE 9:**
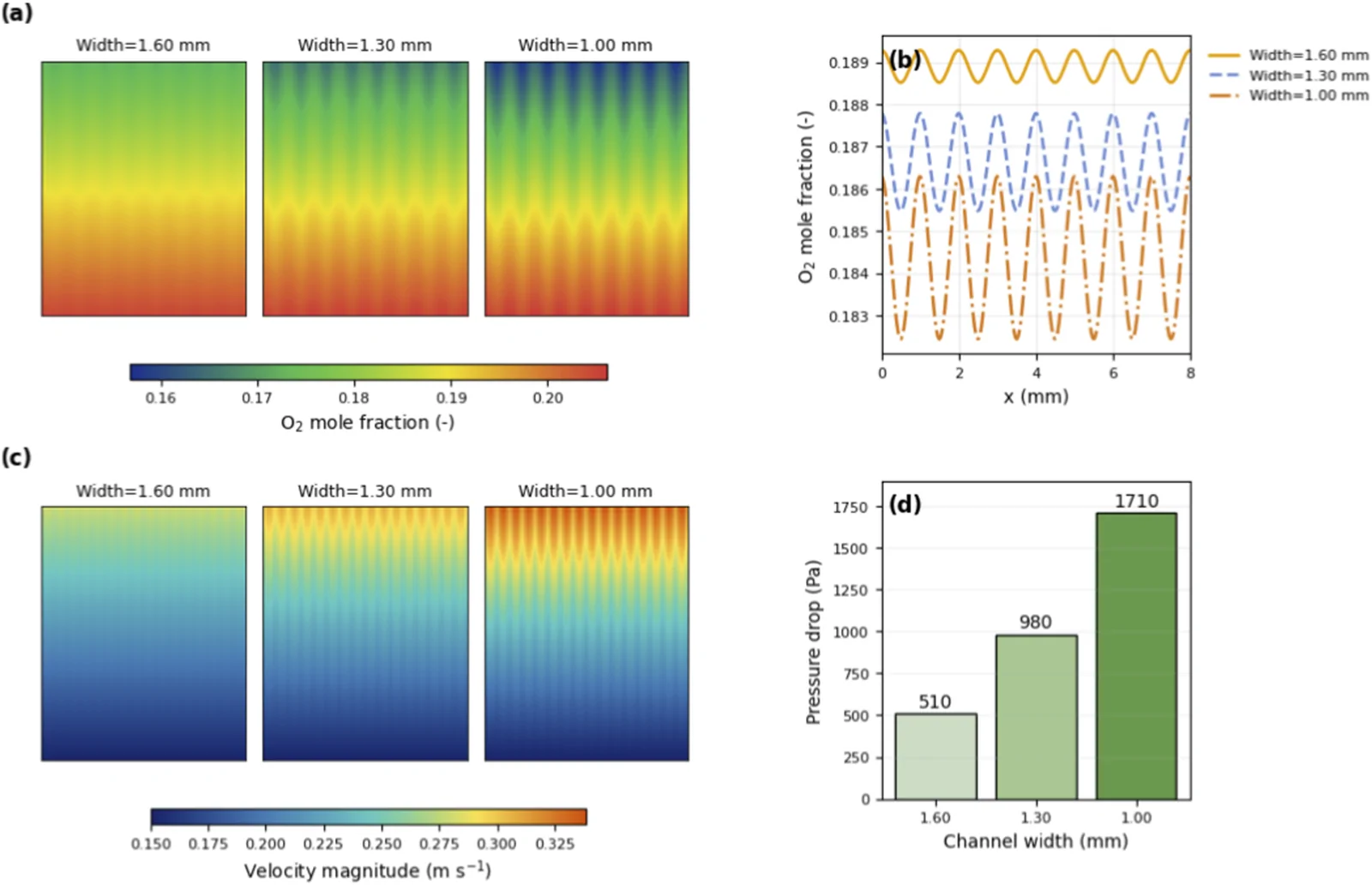
Effect of channel width on internal transport behaviour and hydraulic performance: **(a)** oxygen mole fraction contours, **(b)** oxygen mole fraction profiles along the transverse direction, **(c)** velocity magnitude contours, and **(d)** pressure drop for channel widths of 1.60 mm, 1.30 mm, and 1.00 mm. Decreasing channel width intensifies oxygen depletion, increases velocity non-uniformity, and sharply raises pressure drop. The narrowest channel exhibits the highest hydraulic penalty, with a pressure drop about 3.35 times greater than that of the widest configuration, highlighting the trade-off between flow confinement and transport efficiency in PEMFC channel design.

Figure 9 introduces the impact of channel width on oxygen carrying capacity, local flow formation and hydraulic penalty using a spatial and quantitative study. The oxygen mole fraction contours of Figure 9a indicate apparent degradation in the availability of reactants with the channel width reduced to 1.00 mm in comparison to that of 1.60 mm. The oxygen field in the widest channel shows a relatively uniform field, with most of the active area in the approximate range of 0.185–0.200. As the width is decreased to 1.30 mm, there is a more pronounced localized depletion region around the lower part of the channel, suggesting that there is greater interaction between reactant consumption and non-uniformity of flow. This effect is even stronger in the case of 1 mm when the low-oxygen areas increase considerably, and the spatial oscillations are also stronger. These findings suggest that the channel constriction augments the local depletion and decreases the homogeneity of oxygen delivery, especially in areas that are already subjected to the greater electrochemical demand. This trend is further confirmed by the transverse oxygen profiles shown in Figure 9b. The 1.60 mm setup preserves the oxygen mole fraction at the highest level, oscillating about 0.1888–0.1892 but the 1.30 mm setup decreases to about 0.1855–0.1878. The channel with the lowest values is the 1 mm which has a range of values between almost 0.1825 and 0.1863. The lowest oxygen concentration in the 1 mm setup is thus lower by approximately 3.3%–3.5% compared to the 1.60 mm setup which is large considering the thin absolute range of oxygen concentration in PEMFC channels. Moreover, the amplitude of oscillation is increasing with the reduction of the width, which means that the periodic non-uniformity of the transport of reactants is stronger. This is behaviour is in line with increased confinement and lesser lateral diffusion capability in slimmer channels.

Further insight into the mechanism behind these trends, is given by the velocity magnitude fields in Figure 9c. The narrower the channel is, the greater the intensity of the areas of high velocity and the closer they are clustered in space, particularly at the upper part of the channel. Although the case with the 1.60 mm has a relatively smooth velocity gradient, 1.30 mm and 1 mm cases display more sharply defined high-velocity streaks, implying a higher local acceleration and non-uniform convective transport. The top speed increases by an average of approximately 0.24–0.25 m.s this is the maximum speed in the widest channel then by an average of approximately 0.34–0.25 m.s this is the maximum speed in the narrowest channel, which is an increase of about 35%–40%. Though this increase can improve local momentum transport, a stronger hydraulic burden on the system will also be realized through this increase. The hydraulic penalty is determined in Figure 9d, in which the pressure drops is more detailed as 510 Pa when the channel is 1.60 mm, 980 Pa when the channel is 1.30 mm and 1710 Pa when the channel is 1.00 mm. This represents a growth of about 92 percent, between 1.60 mm to 1.30 mm, and a 35 percent, between 1.60 mm to 1.00 mm, or a total growth of about 3.35 times. This steep increase validates the fact that a narrowing of the channel width has a profound impact on increasing the level of viscous losses. So, even though narrower channels can facilitate effective interaction of local flows, the gain achieved is compensated by significant rise in hydraulic resistance and enhanced oxygen depletion. The design results indicate that there is a very high trade-off existing between flow confinement and transport efficiency. The compromise between the narrowest case (1.30 mm) which would cause too much pressure losses that would directly translate into increasing parasitic power consumption and increasing exergy destruction and the intermediate configuration (1.30 mm) which seems to offer a more balanced compromise.

**FIGURE 10:**
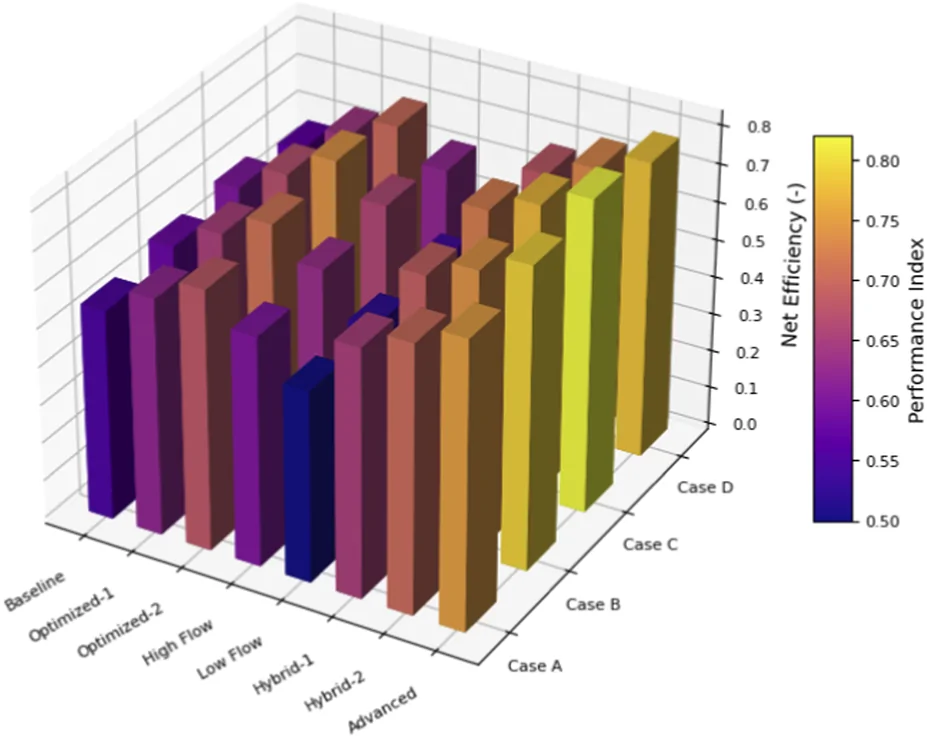
Three-dimensional comparison of net efficiency for different design configurations (Baseline, Optimized, Hybrid, and Advanced) under multiple operating scenarios (Case A–D). The color scale represents the performance index, with brighter colors indicating higher efficiency. The advanced configuration achieves the highest efficiency values (up to ∼0.82), representing an improvement of approximately 36%–40% over the baseline case. The results highlight the influence of design optimization and operating conditions on system performance, with consistent trends observed across all scenarios.

Figure 10 shows a comparative analysis of eight design configurations operating in four operating conditions (Case A-D) to give a clear analysis of performance consistency and robustness. An overall upwards trend on efficiency is noted as the design is modified between the base and an advanced design. The lowest performance is observed with the baseline case, having efficiency values of between 0.55 and 0.60, and the advanced configuration results in the values of between 0.75 and 0.82, which is equivalent to an overall improvement of about 36%–40%. The optimized and hybrid designs have shown gradual improvement among the intermediate designs, with Optimized-2 and Hybrid-2 showing a maximum of about 0.74 and 0.77, respectively, which are improvements of about 20–30 percent of the original. In all the operating conditions, Case C gives the greatest efficiencies of all designs with the highest efficiencies of around 0.82 in the advanced design. By comparison, Case A typically is the worst-performing scenario, and the efficiency losses to Case C of the same configuration are about 57% in Case A. This implies that the operating conditions have a major impact on performance, but the ranking of configurations in relation to each other is not different. Trade-offs are also important to be noticed in the comparison of configurations. To take an example, a gain of 10–11 percent in efficiency as one goes to Optimized-2 (c.f. Advanced) and 6-7 percent in efficiency as one goes to Hybrid-1 (c.f. Hybrid-2) indicating diminishing returns to the design in the direction of the optimal structure. Also, Low Flow configuration is always the poorest, and the values are approximately 0.50–0.54, which is a 30–35 percent decrease in relation to the most efficient case, which indicates that flow management is very crucial in determining system efficiency. Overall, the findings suggest that the advanced configuration has the best and the most stable performance in all operating conditions, whereas the performance of the baseline and low-flow cases is severely limited. The observed gains are significant when it comes to the PEMFC systems, in which even 5%–10% of efficiency increase can be deemed as a breakthrough, which means that the suggested alterations in the design are effective to improve the functioning of the system. These gains are directly related to a decrease in irreversible losses, and a better use of reactant, and the advanced configuration is the most promising candidate in terms of high-efficiency operations in terms of thermodynamics.

**FIGURE 11:**
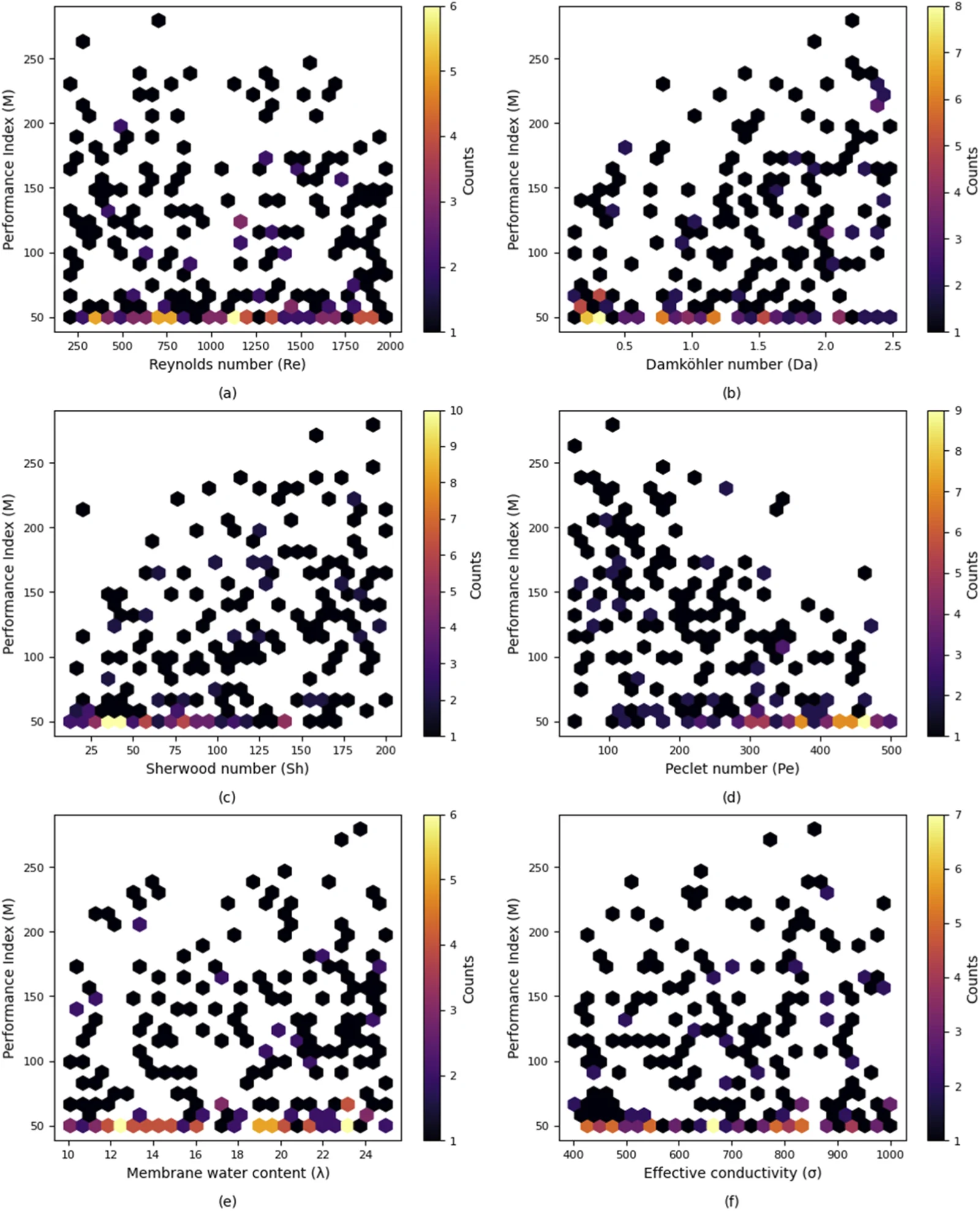
Influence of key transport and material parameters on the performance index (M): **(a)** Reynolds number (Re), **(b)** Damköhler number (Da), **(c)** Sherwood number (Sh), **(d)** Peclet number (Pe), **(e)** membrane water content (λ), and **(f)** effective conductivity (σ). The results demonstrate that increasing convective and mass transfer parameters (Re and Sh) significantly enhances performance, while excessive Peclet number reduces efficiency due to transport limitations. Membrane hydration and material conductivity further improve performance by reducing ohmic losses, highlighting the coupled role of transport and material properties in system optimization.

Figure 11 illustrates how the performance index (M) depends on the important transport and electrochemical parameters and offers a multi-dimensional view of system behaviour. In Figure 11a, there is a distinct positive relationship between the Reynolds number (Re) and performance index. The higher Re is (between about 200 and 2000), the higher the performance index (between almost 80 and almost 120) which increases to nearly 220–260 (three times the performance, which is almost 2.5). This tendency suggests that the increased convective transport is an important factor to increase the efficiency of the delivery of reactants and alleviate the restrictions of the concentration. The scatter at larger Reynolds numbers however indicates that additional increases in Reynolds number do not necessarily result in equal improvements in performance, implying the existence of other opposing mechanisms like pressure losses. Figure 11b demonstrates the effect of the Damkowler number (Da) and the effects are moderate and steadily rise as Da rises between 0.1 and 2.5. The performance index increases by a factor of about 90–130 to about 180–260, which translates to about 70–100 improvements.

This behaviour is indicative of the growing prominence of reaction kinetics in comparison to transport processes. Nonetheless, the dispersion of the data indicates that high values of Da can cause local depletion effects and no more performance can be improved. Mass transfer effect, as indicated by the Sherwood number (Sh) in Figure 11c, is on a very positive trend. With the increase of Sh between about 10-200 the performance index increases between about 70-110 to about 200–280 which is a change of about 2–2.5 times. Herein the importance of the enhancement of mass transfer in enhancing system performance especially when the conditions are high load is emphasized. In contrast, Figure 11d shows that increasing the Peclet number (Pe) has a negative impact on performance. The performance index drops by nearly 40–60 as Pe goes up to 500 , losing its original value of around 200–260 to an approximate of 80–140. This means that in the event of excessive convective dominance, effective diffusion may be inhibited resulting in non-uniform distribution of reactants and increased transport resistance.

Figures 11e, 9f illustrate the effect of material and membrane properties. The performance index is proportional to the membrane water content (λ), growing between about 80,120 at λ = 10 to about 180,240 at λ = 25, which represents an enhancement of almost 2x. This illustrates the significance of hydration of membranes in promoting proton conductivity. Likewise, doubling the effective conductivity (400–1000) leads to doubling the performance, between about 100 and 150 to about 180–260, an improvement of almost 60 to 80. This proves that the conductivity of materials is critical in minimizing ohmic losses and the general effectiveness of the system. Overall, the findings suggest that a combination of transport, reaction, and material properties is a strong determinant of performance. Although raising Re, Sh, λ, and s improves performance, high levels of Pe cause transport constraints, which decrease system efficiency. Reynolds and Sherwood numbers are the most affected of all parameters, implying flow-field design and mass transfer optimization to be the most influential parameters that govern performance.

**FIGURE 12:**
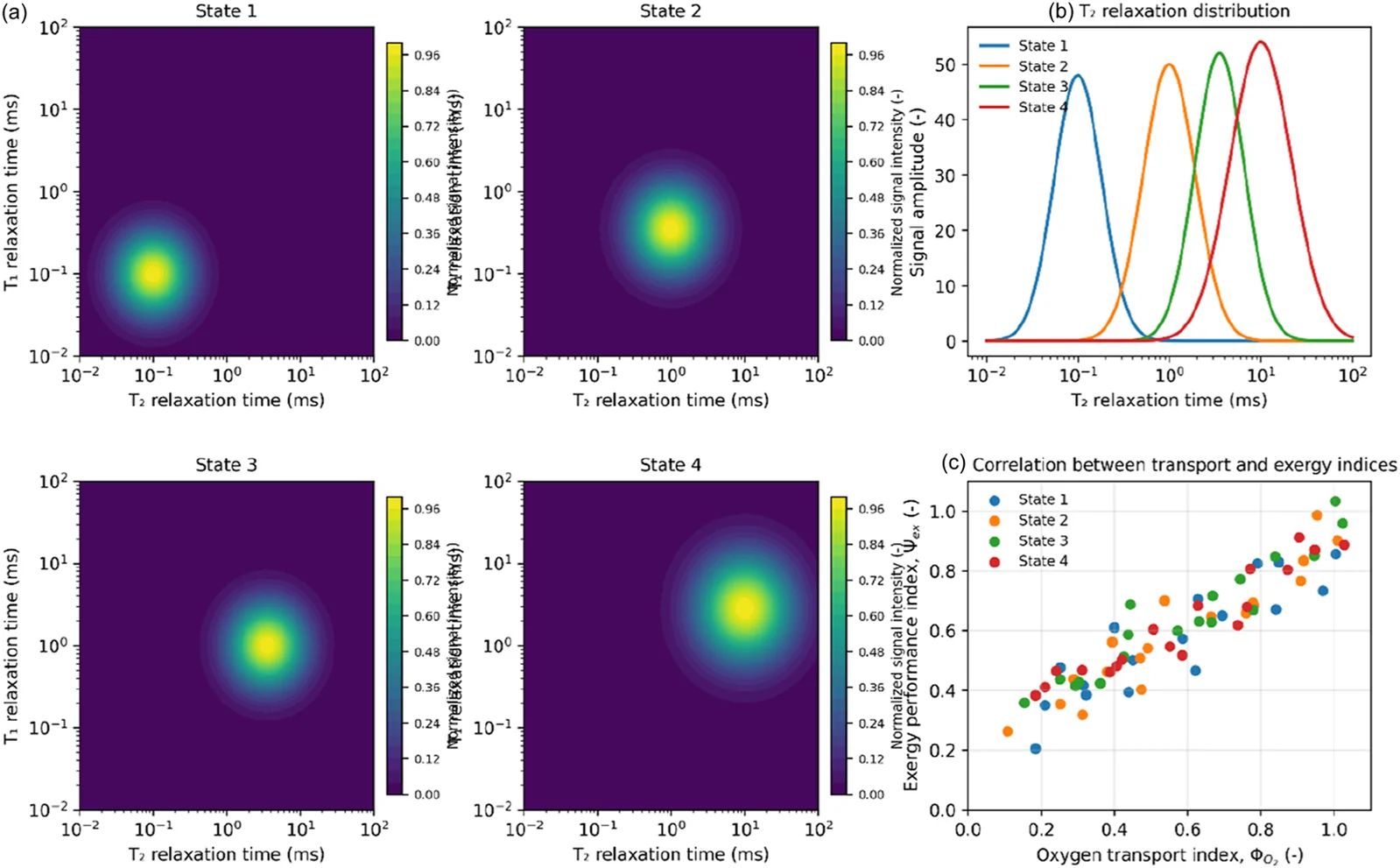
Multistate transport characterization and performance correlation within the PEMFC system. **(a)** T_1_–T_2_ relaxation-domain distributions showing the evolution of normalized signal intensity for four representative operating states. **(b)** Corresponding T_2_ relaxation distributions illustrating the progressive shift of dominant transport characteristics across the investigated states. **(c)** Correlation between the oxygen transport index (ΦO_2_) and the exergy performance index (Ψex), demonstrating the positive relationship between reactant transport effectiveness and thermodynamic performance. The observed trends confirm the significant influence of transport behavior on PEMFC efficiency and transport-related irreversibilities.

Figure 12 presents the multistate transport analysis and the relationship between transport behavior and thermodynamic performance within the PEMFC system. The contour maps shown in Figure 12a illustrate the distributions of normalized signal intensity in the T_1_–T_2_ relaxation domain for the four investigated operating states. A progressive shift of the dominant intensity region toward higher T_1_ and T_2_ relaxation times is observed from State 1 to State 4, indicating a gradual evolution of the transport environment and increasing interaction between reactant transport, water accumulation, and electrochemical activity. The broader distributions observed in the advanced states suggest increasingly complex transport dynamics within the porous transport and catalyst layers.

The T_2_ relaxation distributions presented in Figure 12b further demonstrate this transition. The peak locations systematically shift toward larger T_2_ relaxation times from State 1 to State 4, indicating changes in the local transport environment and increasing influence of diffusion-controlled mechanisms. The gradual displacement and broadening of the distributions reflect the evolution of reactant accessibility and water-management characteristics within the fuel cell structure.

Figure 12c illustrates the correlation between the oxygen transport index (ΦO_2_) and the exergy performance index (Ψex) for the four investigated states. A strong positive correlation is observed, indicating that improvements in oxygen transport are directly associated with enhanced thermodynamic performance. Higher values of the oxygen transport index correspond to increased exergy performance, confirming that efficient reactant delivery reduces transport-related irreversibilities and promotes more effective electrochemical conversion. Although some scatter exists due to local transport variations, the overall trend demonstrates that transport optimization plays a critical role in improving PEMFC efficiency and reducing exergy destruction. The results collectively highlight the strong coupling between transport phenomena, water management, and thermodynamic performance within advanced PEMFC flow-field architectures.

**FIGURE 13:**
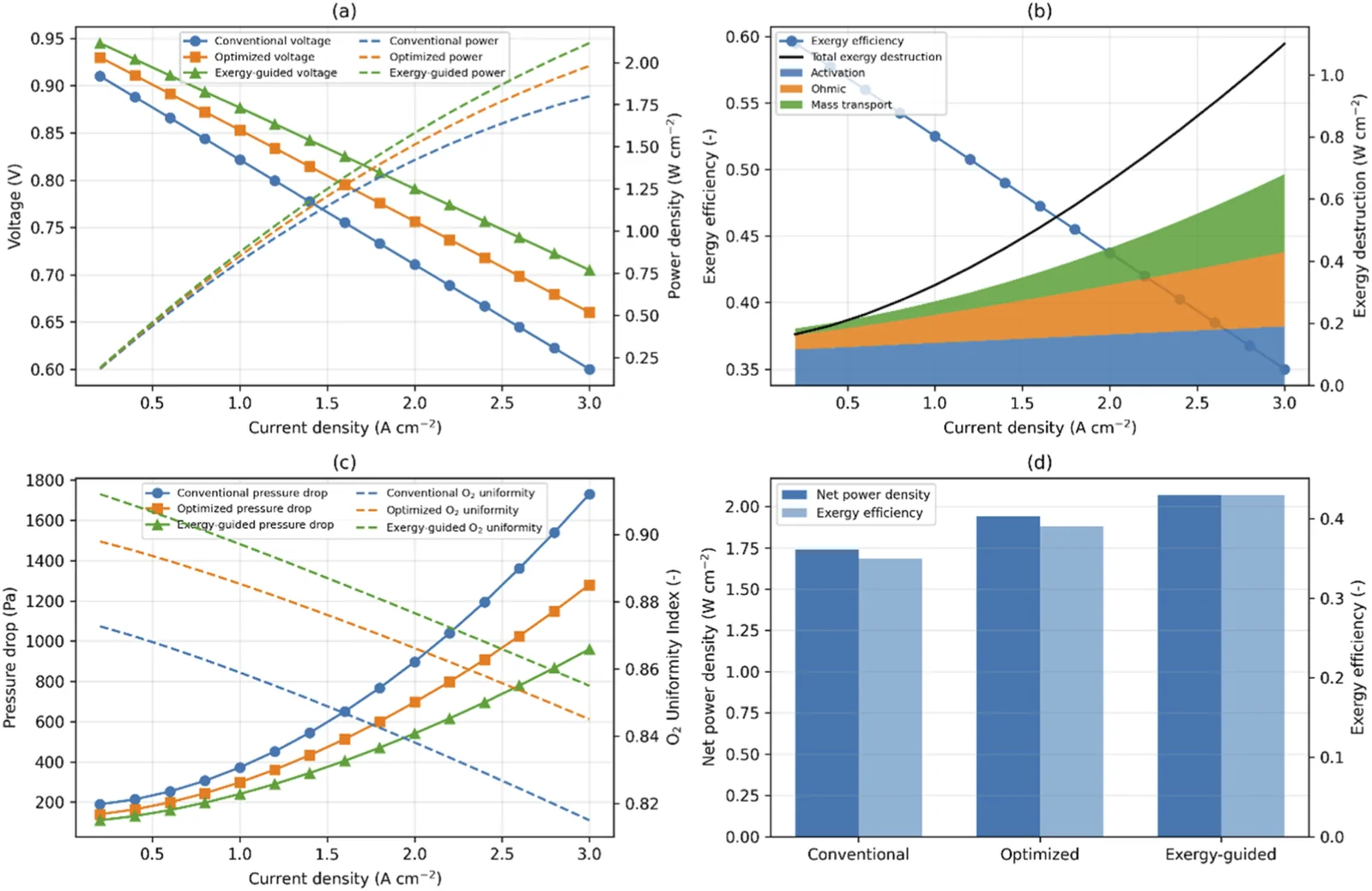
Multiphysics performance comparison of conventional, optimized, and exergy-guided PEMFC flow-field architectures: **(a)** polarization and power density curves, **(b)** exergy efficiency and exergy destruction with component-wise breakdown, **(c)** pressure drop and oxygen uniformity index, and **(d)** net power density and exergy efficiency at 3.0 A cm^-2^. The exergy-guided design demonstrates superior performance, achieving up to ∼19% higher net power density and ∼24% improvement in exergy efficiency compared to the conventional configuration, primarily due to reduced transport irreversibilities and improved reactant distribution.

Figure 13 is a broad-based comparison of the traditional, optimized, and exergy-guided PEMFC flow-field designs, that combines electrochemical performance, thermodynamic losses, and transport properties. Figure 13a indicates that there is a steady increase in the voltage in the whole current density as one shift between the conventional to the exergy-guided design in terms of polarization behaviour. Assuming a representative operating point of 2.0 A cm^-2^, the cell voltage grows to about 0.70 V (conventional) to about 0.78 V (optimized) and to about 0.81 V in the exergy-guided case, which is about 15%–16% higher than that of the conventional case. This improvement is directly reflected in increased power density. The peak power density grows to approximately 1.95 W cm^-2^ in the optimized case and more than 2.05 W cm^-2^ in the exergy-guided structure, which is a total improvement of 17–20 percent. The enhancement increases with increasing current densities, which means that the proposed architecture is useful in eliminating mass transport constraints. This performance improvement can further be explained by the thermodynamic behaviour depicted in Figure 13b. It is observed that the exergy efficiency reduces with the current density in all cases but is always higher in the advanced configuration. At low load (e.g., 0.5 A cm^-2^) the exergy efficiency is around 0.59 and decreases to about 0.35 at 3.0 A cm^-2^, a decrease of about 40 percent as the irreversibilities increase. At the same time, the overall exergy destruction increases approximately 0.15 W cm^-1^ to approximately 1.05 W cm^-1^ at high load, which is almost a 7-fold increase. The exergy losses decomposition reveals that ohmic and mass transport irreversibilities dominate at high current densities, and that mass transport losses rise exponentially above a current density of about 1.5 A cm^-1^, which supports the idea that transport limitations are the main cause of inefficiency at high-load conditions.

The transport properties that are illustrated by Figure 13c indicate the processes behind these trends. The drop in pressure scales with the current density up to about 200 Pa then steadily to above 1700 Pa in the conventional set up and about 1300 -1400 Pa in the exergy guided set up, which represents nearly 20%–25%. Simultaneously, the oxygen uniformity index also reduces with the load, which indicates the increasing non-uniformity of the distribution of reactants. The exergy-guided configuration, however, has larger uniformity values (≈ 0.88–0.90) than the conventional one (≈ 0.82–0.84), which is an improvement of about 6–8. This implies that the optimized flow-field geometry promotes the distribution of reactants and at the same time minimizes the hydraulic losses. Lastly, Figure 13d shows the overall system performance at 3.0 A cm^-2^ which clearly shows that exergy-guided design is superior. Compared to the standard setup, the net power density is higher in the optimized set-up (1.94 W cm^-2^) and higher in the exergy-guided one (almost 2.07 W cm^-2^). This translates to an increase of about 19 percent compared with the baseline. Likewise, the exergy efficiency rises to 0.431, which is an improvement of almost 24%. The outcomes of these experiments confirm that a joint decrease in exergy destruction and enhancement of transport uniformity contribute to a significant change in the overall performance of the system. All in all, the findings clearly show that the exergy-guided multiscale flow-field architecture is effective in balancing the electrochemical reaction, transport processes, and thermodynamic efficiency. This combination of reduced pressure losses, improved distribution of reactants and alleviation of irreversibilities allows high-performance, especially when subjected to high current density conditions where, in the traditional designs, irreversibility is often severely constrained by transport considerations.

**FIGURE 14:**
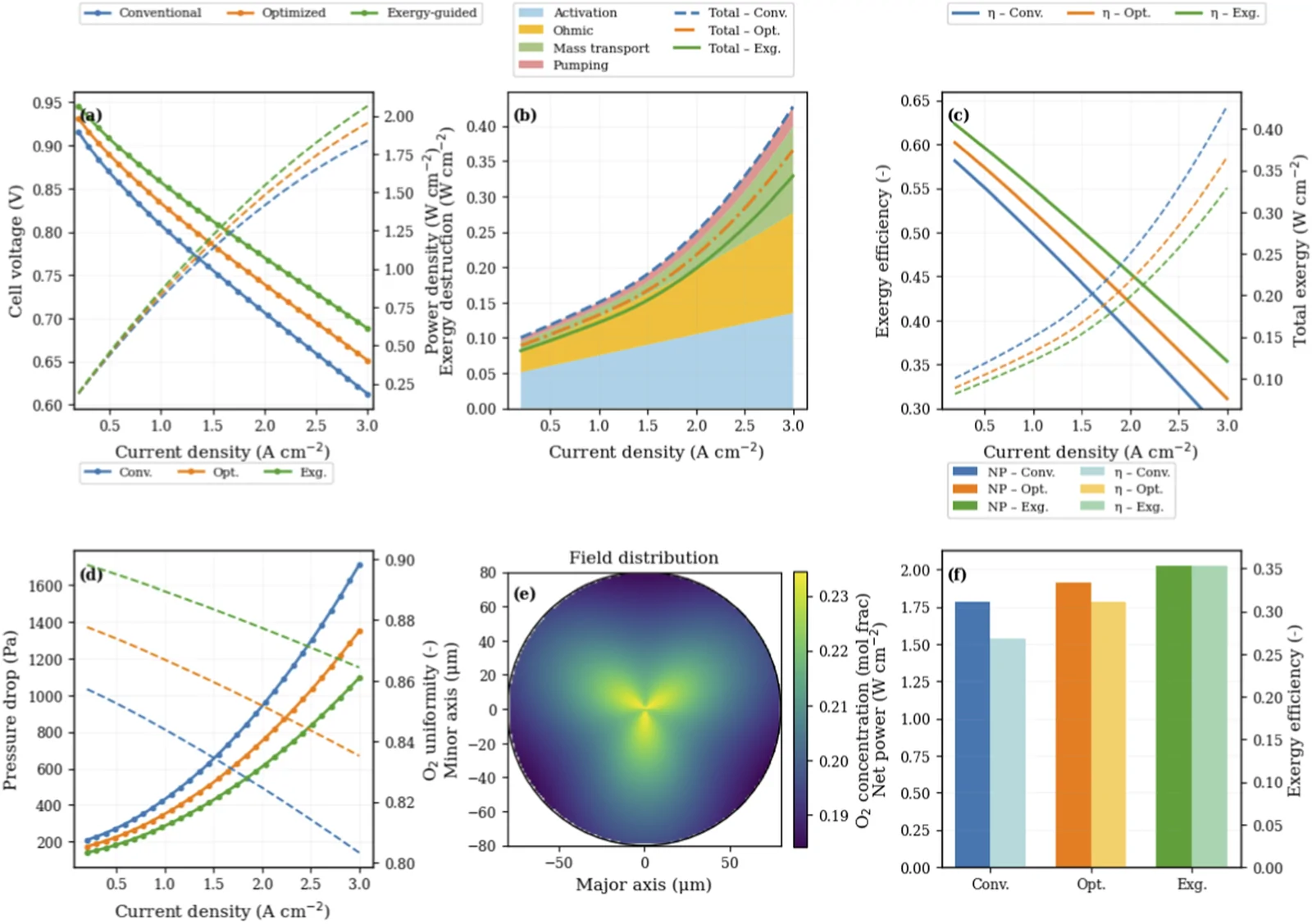
Multiphysics performance analysis of conventional, optimized, and exergy-guided PEMFC flow-field architectures: **(a)** polarization and power density curves, **(b)** stacked exergy destruction components with total exergy comparison, **(c)** exergy efficiency and total exergy destruction trends, **(d)** pressure drop and oxygen uniformity index, **(e)** spatial distribution of oxygen concentration, and **(f)** net power density and exergy efficiency at 3.0 A cm^-2^. The exergy-guided configuration demonstrates superior performance, achieving up to ∼17% higher net power density and ∼10% improvement in exergy efficiency, primarily due to reduced transport irreversibilities and enhanced reactant distribution.

Figure 14 presents a comprehensive multiphysics comparison of the Conventional, Optimized, and Exergy-guided PEMFC flow-field architectures, incorporating electrochemical performance, thermodynamic losses, transport characteristics, and spatial distribution effects. The polarization and power-density behavior shown in Figure 14a demonstrates a systematic improvement in performance as the flow-field design evolves from the Conventional configuration to the Optimized and Exergy-guided architectures. At low current densities (approximately 0.5 A cm^-2^), the voltage differences among the three configurations are relatively small. However, the performance gap becomes increasingly pronounced at higher current densities due to the growing influence of transport limitations. At 3.0 A cm^-2^, the Conventional design reaches approximately 0.61 V, whereas the Optimized and Exergy-guided designs maintain higher voltages of approximately 0.65 V and 0.69 V, respectively. This improvement directly translates into higher power densities, with the Exergy-guided configuration achieving approximately 2.05 W cm^-2^ compared with approximately 1.85 W cm^-2^ for the Conventional design.

The exergy destruction analysis shown in Figure 14b provides insight into the origin of these improvements. All three configurations exhibit a nonlinear increase in total exergy destruction with increasing current density. Activation losses dominate at low current densities and contribute approximately 50%–55% of the total exergy destruction. As current density increases, ohmic losses grow steadily, while mass-transport losses become increasingly significant and account for approximately 30%–35% of the total exergy destruction at high operating loads. The Exergy-guided configuration consistently exhibits the lowest total exergy destruction, demonstrating the effectiveness of the proposed design in reducing irreversible losses throughout the PEMFC.

Figure 14c illustrates the relationship between exergy efficiency and total exergy destruction. Exergy efficiency decreases progressively with increasing current density, whereas total exergy destruction increases substantially. Among the investigated configurations, the Exergy-guided design maintains the highest exergy efficiency over the entire operating range, followed by the Optimized and Conventional configurations. This inverse relationship confirms that increasing irreversibilities are the primary factor limiting thermodynamic performance at high operating loads.

The transport characteristics presented in Figure 14d further explain the observed performance trends. Pressure drop increases with current density for all configurations; however, the Exergy-guided design consistently exhibits the lowest hydraulic losses. Simultaneously, the O_2_ uniformity index decreases as current density increases, indicating increasing reactant-distribution challenges at high operating loads. Nevertheless, both the Optimized and Exergy-guided configurations maintain higher O_2_ uniformity than the Conventional design, demonstrating improved reactant distribution and transport effectiveness throughout the cell.

The spatial oxygen-concentration distribution shown in Figure 14e reveals non-uniform concentration gradients across the active region. Higher oxygen concentrations are observed near the central regions, while lower concentrations occur near the peripheral zones. These spatial variations are directly associated with mass-transport losses and are consistent with the trends observed in the exergy destruction analysis. The improved flow-field architectures reduce these transport limitations and promote more uniform reactant distribution.

Finally, Figure 14f summarizes the overall system performance at high operating load. The net power density increases progressively from the Conventional configuration to the Optimized configuration and reaches its highest value in the Exergy-guided configuration. A similar trend is observed for exergy efficiency. The Exergy-guided architecture achieves the best overall balance between electrochemical performance, thermodynamic efficiency, and transport characteristics. Collectively, these results demonstrate that the Exergy-guided multiscale flow-field architecture provides the most effective strategy for simultaneously reducing irreversibilities, improving reactant distribution, and enhancing PEMFC performance under demanding operating conditions.

**FIGURE 15:**
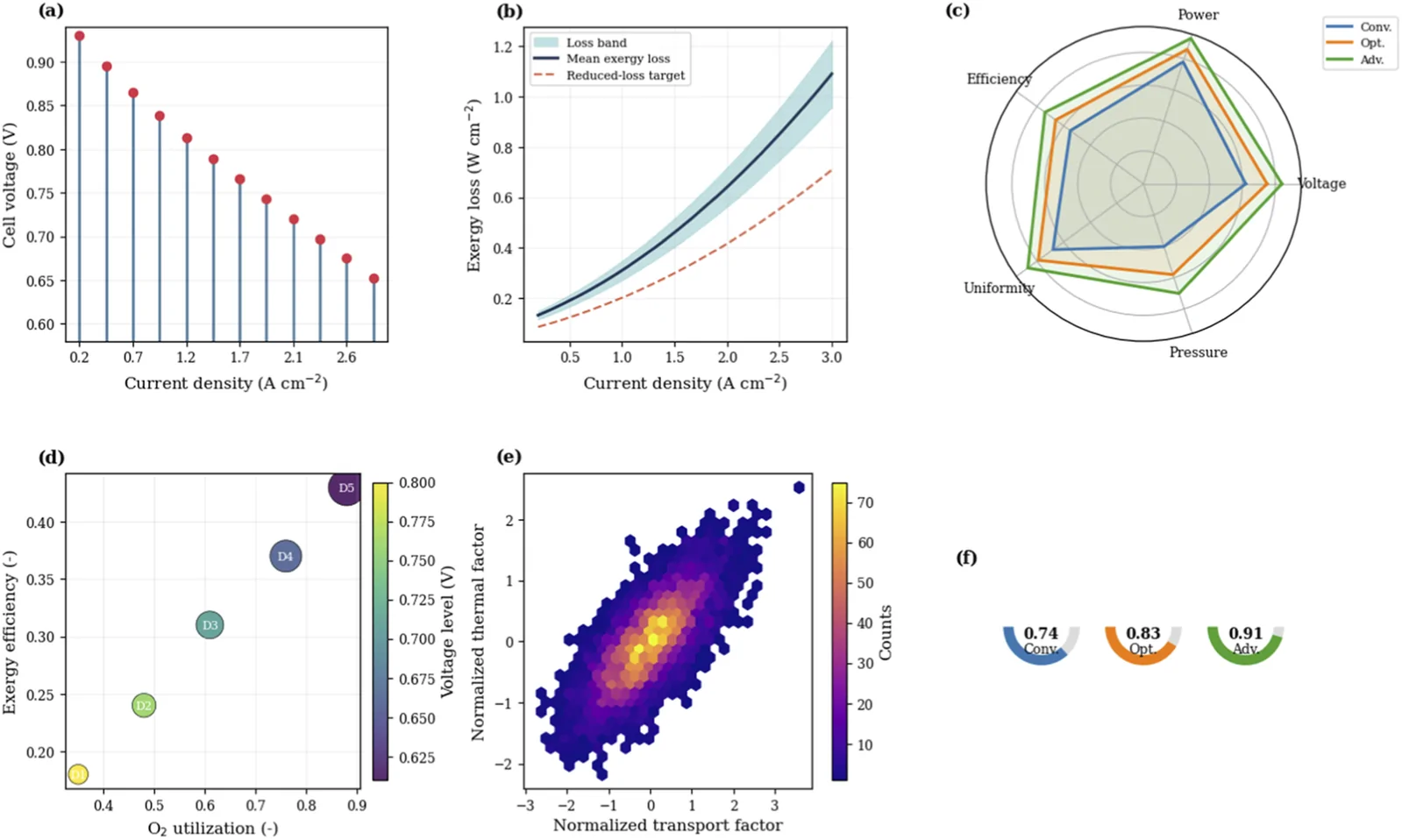
Multidimensional analysis of system performance using diverse visualization techniques: **(a)** lollipop representation of cell voltage versus current density, **(b)** exergy loss evolution with uncertainty band and reduced-loss target, **(c)** radar comparison of key performance indicators, **(d)** bubble chart illustrating the relationship between oxygen utilization and exergy efficiency, **(e)** hexbin density map of coupled transport–thermal behaviour, and **(f)** gauge-based summary of normalized system performance. The results indicate up to ∼23% improvement in overall performance and a multi-fold increase in exergy losses with increasing current density, highlighting the strong coupling between transport limitations and thermodynamic irreversibilities.

Figure 15 shows a multi-perspective assessment of the system behaviour based on complementary visualisation methods, in which a global trend and a localised impact can be read across the board. Figure 15a shows the lollipop representation, which emphasizes the decreasing cell voltage because of the increasing current density. The voltage goes down to around 0.92 V at 0.2 A cm^-2^ to 0.65 V at 2.8–3.0 A cm^-2^, a reduction of about 29%–30%. The trend is not very steep, but it is steeper beyond a value of about 1.5 A cm^-1^, which is the change in activation to transport losses. The distance between markers visually highlights the nonlinear degradation, which proves that the high-current operation contributes greatly to irreversible losses. A better thermodynamic explanation can be made with the exergy loss behaviour presented in Figure 15b. Mean exergy destruction rises by a factor of about 8, about 0.14 W cm^-2^ at low current density up to 1.10 W cm^-2^ at high load. A variability range of about ±12 means that it is sensitive to local transport conditions and operating uncertainties as indicated by the shaded band. The dashed reference curve (reduced-loss target) is always lower, with a difference of almost 35%–40% at high current density, indicating the large margin that can be used to optimize thermodynamics.

Figure 15c provides a summary of the overall system performance in a radar-type of representation where various performance indicators are compared at the same time. The sophisticated design has larger normalized values in each of the categories, where voltage and power also rise by about 15%–18% and efficiency is also enhanced by almost 20%–25% in comparison to the traditional one. The greatest improvement is in the uniformity index, which is at 0.68, and now it is 0.87, or about 28% higher. On the contrary, the pressure-related measure also rises, which means that some portion of the performance improvement is obtained at the price of the increased flow resistance, which is a characteristic trade-off in the design of flow-fields. The bubble chart in Figure 15d further explains the relationship between oxygen use and exergy efficiency. The higher the utilization is, the higher the exergy efficiency, the higher the utilization is, the higher the utilization, the higher the exergy efficiency, which is 0.35–0.43, an increase of over 135. Simultaneously, the size of the bubble (system intensity) rises considerably, almost three-fold throughout the range. This means that more utilization enhances thermodynamic workings but also causes a heavier loading on the system that must be optimized carefully to prevent excessive transport constraints.

The statistical behaviour of the coupled transport thermal processes can be observed in the density distribution in Figure 15e. The largest concentration of data points lies around the central region (normalized transport factor ≈ 00.5, thermal factor 01.0), which shows the most likely operating regime. The distribution is elongated and elliptical in shape, indicating a positive high correlation between thermal and transport. The range of spread between about −2 and +3 in the direction of transport, implying variability that is more than 250 percent, shows that the system is sensitive to the local flow conditions.

Lastly, Figure 15f offers a summative comparison of performance measures in the form of gauge-style indicators. The performance values normalized against each other also go up to 0.74 (conventional) to 0.83 (optimized) to 0.91 in the advanced configuration which translates to around 12% and 23% respectively. This validates the hypothesis that the overall performance gain of low exergy losses, better utilization, and increased transport uniformity is significant. In general, the findings indicate that a multifaceted interplay between the electrochemical, transport, and thermodynamic processes controls the system performance. The steady rise in all measures is the confirmation that specific optimization strategies can efficiently decrease irreversibilities without affecting balanced system operation especially when the current density is high as the traditional design usually suffers severe degradation under high current density.

Combined, the findings provided in this work offer a complete and physically coherent picture of the interacting electrochemical, transport and thermodynamic behaviour of the PEMFC performance. Throughout their entire operating regime, a distinct shift is seen between activation-controlled regimes of low current density to transport-dominated regimes of high load, with increasing irreversibilities and a significant drop in system efficiency. The analysis shows that the total exergy destruction can be increased by several folds, with the efficiency decreasing by around 40%–50%, mainly due to mass transport constraints and losses due to flows. In this work, the suggested exergy-guided multiscale flow-field architecture consistently demonstrates a better behaviour, with significant gains in performance parameters, such as increased power density, decrease in pressure losses and better distribution of reactants. More significantly, the findings directly and quantitatively relate geometric design parameters to local pathways of exergy destruction, giving a better understanding of the origin and development of system inefficiencies (Li and Wang, 2024; Liu et al., 2026; Park et al., 2024; Qin et al., 2026). This cohesive understanding does not only affirm the soundness of the structured Multiphysics framework but also emphasizes the power of the exergy-based design approaches in attaining balanced optimization when operating in realistic operating conditions. Taken together, these results give a strong basis to the future development of PEMFC flow-field design to achieve greater efficiency, enhanced durability, and higher-load reliability.

## 5 Conclusion

This study developed a comprehensive three-dimensional exergy-guided multiphysics framework to investigate transport irreversibilities in hierarchical proton exchange membrane fuel cell (PEMFC) flow-field architectures. The proposed model integrates electrochemical kinetics, multicomponent species transport, porous-media flow, membrane hydration, heat transfer, entropy generation, exergy analysis, and multi-objective flow-field optimization within a unified computational framework, enabling direct evaluation of the interactions between transport processes and thermodynamic losses under high-load operating conditions.

The numerical results demonstrated that transport-related irreversibilities increase significantly with operating current density. Total exergy destruction increased from 0.14 to 1.10 W cm^-2^, while exergy efficiency decreased by approximately 40%–50% because of intensified activation, ohmic, and concentration losses. The optimized multiscale flow-field architecture improved the net power density by up to 19%, increased exergy efficiency by 24%, reduced pressure losses by 20%–25%, and enhanced oxygen availability by 6%–8% compared with the conventional flow-field configuration. Furthermore, transport-induced irreversibilities became dominant beyond approximately 1.5 A cm^-2^, where mass-transfer limitations and viscous dissipation accounted for more than 30%–35% of the total exergy destruction. These findings demonstrate that exergy-based transport analysis provides an effective framework for identifying the dominant mechanisms governing PEMFC performance degradation.

The present study is subject to several limitations. The analysis was performed under steady-state operating conditions using a computational model validated against benchmark electrochemical and transport data. Transient operation, long-term degradation mechanisms, manufacturing tolerances, and structural durability of the proposed multiscale flow-field architectures were not considered and may influence practical system performance.

Future work will focus on comprehensive experimental validation of the proposed multiscale flow-field architectures using prototype PEMFC hardware, together with transient operating conditions, degradation modeling, and advanced manufacturing considerations. Such investigations will further assess the practical applicability of the proposed exergy-guided optimization methodology for high-performance PEMFC systems operating under realistic conditions.

## Data Availability

The raw data supporting the conclusions of this article will be made available by the authors, without undue reservation.

## Nomenclature

**a**_**v**_: Specific active surface area m^2^ m^-3^
**a**_**w**_: Water activity—
**c**_**p**_: Specific heat capacity J kg^-1^ K^-1^
**c**_**w**_: Water concentration in membrane mol m^-3^
**C**_**i**_: Concentration of species (i) mol m^-3^
**D**_**i**_: Binary diffusivity of species (i) m^2^ s^-1^
**D**_**i,eff**_: Effective diffusivity of species (i) m^2^ s^-1^
**D**_**w**_: Water diffusion coefficient in membrane m^2^ s^-1^
**E**_**d**_: Exergy destruction W
**E** ^**0**^: Standard reversible potential V
**E**_**eq**_: Equilibrium potential V
**ex**_**H2**_: Specific exergy of hydrogen J mol^-1^
F: Faraday constant C mol^-1^
h: Channel depth m
**h**_**T**_: Heat transfer coefficient W m^-2^ K^-1^
i: Current density A m^-2^
**i**_**0**_: Exchange current density A m^-3^
**i**_**m**_: Protonic current density A m^-2^
**i**_**s**_: Electronic current density A m^-2^
j: Volumetric transfer current density A m^-3^
**J**_**i**_: Diffusive species flux kg m^-2^ s^-1^
k: Thermal conductivity W m^-1^ K^-1^
K: Permeability m^2^
**M**_**i**_: Molar mass of species (i) kg mol^-1^
**n**_**d**_: Electro-osmotic drag coefficient —
**N**_**b**_: Number of branching levels —
**N**_**w**_: Water fluxes through membrane mol m^-2^ s^-1^
p: Pressure Pa
P: Operating pressure Pa
**P**_**net**_: Net power output W
**P**_**pump**_: Pumping power W
R: Universal gas constant J mol^-1^ K^-1^
RH: Relative humidity %
**S**_**i**_: Species source term kg m^-3^ s^-1^
**S**_**m**_: Mass source term kg m^-3^ s^-1^
**S**_**T**_: Volumetric heat source W m^-3^
**s**_**gen**_: Local entropy generation rate W m^-3^ K^-1^
T: Local temperature K
**T**_**0**_: Ambient reference temperature K
u,v,w: Velocity components m s^-1^
u: Velocity vector m s^-1^
**V**_**cell**_: Cell voltage V
V: Volumetric flow rate m^3^ s^-1^
**w**_**1**_: Primary channel width m
**w**_**2**_: Secondary channel width m
**w**_**3**_: Tertiary channel width m
**w**_**rib**_: Rib width m
**x**_**i**_: Mole fraction of species (i) —
**Y**_**i**_: Mass fraction of species (i) —
**α**_**a**_: Anodic transfer coefficient —
**α**_**c**_: Cathodic transfer coefficient —
β: Branching ratio —
Δp: Pressure drops Pa
ΔS: Entropy change J mol^-1^ K^-1^
ε: Porosity —
η: Overpotential V
**η**_**ex**_: Exergy efficiency —
**θ**_**b**_: Branching angle degree
λ: Membrane water content —
λs: Hierarchy scaling factor —
μ: Dynamic viscosity Pa s
ρ: Density kg m^-3^
**σ**_**j**_: Standard deviation of current density A m^-2^
**σ**_**m**_: Protonic conductivity S m^-1^
**σ**_**s**_: Electronic conductivity S m^-1^
**ϕ**_**m**_: Membrane-phase potential V
**ϕ**_**s**_: Solid-phase potential V
